# Rotating Gold Nanomotors for High‐Resolution Mapping of Subcellular Nanomotions

**DOI:** 10.1002/smtd.202501393

**Published:** 2025-10-21

**Authors:** Emelie Tornéus, Charlotte Hamngren Blomqvist, Caroline Beck Adiels, Hana Šípová‐Jungová

**Affiliations:** ^1^ Department of Physics Chalmers University of Technology 412 96 Göteborg Gothenburg Sweden; ^2^ Department of Physics University of Gothenburg 412 96 Göteborg Gothenburg Sweden

**Keywords:** cellular nanomotion, mechanophenotyping, optical trapping, plasmonic nanorods, single‐cell analysis

## Abstract

Nanometer‐scale movements (nanomotions) of living cells provide sensitive indicators of cellular state and viability, yet capturing such dynamics with nanometer precision and subcellular resolution remains a major challenge. To overcome this limitation, a light‐driven nanomotor platform based on plasmonic gold nanorods is introduced, in which circularly polarized light traps and rotates individual nanorods to transduce local cellular nanomotions into measurable rotational‐frequency fluctuations. The frequency‐based readout achieves tunable performance, offering 10 nm axial precision at second timescales, or 130 nm precision with millisecond temporal resolution over ≈300 × 300 nm^2^ regions of the cellular surface. Applied to human microvascular endothelial cells (HMEC‐1), the method resolves heterogeneous nanomotion patterns across the nucleus, perinuclear region, and cell periphery. The measured nanomotion amplitudes greatly exceed frequency fluctuations observed on glass and fixed cells, confirming that the signals originate from active cellular processes. Transient oscillations at 10–20 Hz are detected in nuclear and perinuclear regions, revealing short‐lived mechanical events that are typically inaccessible to conventional methods. Power spectral analysis further uncovers scale‐invariant 1/f^α^ dynamics, distinguishing correlated and stochastic motion regimes. Together, these results establish a label‐free and non‐invasive approach for quantitative, high‐resolution mapping of subcellular mechanics and dynamic processes in living cells.

## Graphical abstract

A nanomotor‐based method uses optically driven, rotating gold nanorods to detect nanometer‐scale cellular movements with 10 nm spatial and millisecond temporal resolution. Applied to live endothelial cells, the technique reveals subcellular mechanical activity and distinct frequency regimes, capturing transient, localized oscillations. This label‐free approach enables probing of dynamic cellular processes in healthy and diseased states.

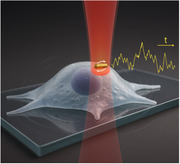

## 1 Introduction

Single cells exhibit nanoscale movements that reflect their physiological state and mechanical properties.^[^ ^1^ ^]^ These movements span a broad spectrum, from nanometer‐scale protein displacements to coordinated oscillations across entire cell populations.^[^ ^2^, ^3^ ^]^ At the cellular level, they are driven by key biological processes,^[^ ^4^ ^]^ which collectively support cell growth, metabolism,^[^ ^5^ ^]^ and division.^[^ ^6^, ^7^ ^]^ Alterations in the amplitude, frequency, or spatial distribution of these motions can reflect pathological changes in cytoskeletal integrity, energy metabolism, or membrane mechanics, hallmarks of numerous diseases, including cancer.^[^ ^8^ ^]^ As early mechanical biomarkers, nanomotions offer valuable insight into cellular viability^[^ ^9^, ^10^ ^]^ and the onset of disease. However, capturing these transient, nanoscale events in living cells with sufficient spatial and temporal resolution remains a significant experimental challenge.

Efforts to detect these nanomotions have led to a variety of techniques aimed at assessing cell viability and mechanical behavior. Surface‐based methods, such as atomic force microscopy (AFM),^[^ ^10^, ^11^, ^12^ ^]^ quartz crystal microbalance (QCM),^[^ ^13^ ^]^ plasmonic sensors,^[^ ^14^, ^15^ ^]^ impedance sensing (IS),^[^ ^16^, ^17^, ^18^ ^]^ and graphene drums,^[^ ^19^ ^]^ capture mechanical signals by transferring cellular oscillations to a sensor surface. These signals vanish when viability is lost,^[^ ^20^, ^21^ ^]^ offering a global but coarse indication of cell activity. Moreover, these methods typically operate at the whole‐cell or population level, often masking the contributions of subcellular structures by blending them into background noise.

Optical techniques, such as phase imaging^[^ ^22^, ^23^ ^]^ and digital holographic microscopy,^[^ ^24^ ^]^ provide non‐invasive alternatives, offering improved spatial resolution without physical contact. However, their resolution remains diffraction‐limited (200–300 nm), and they are most effective in optically homogeneous cells, such as red blood cells.^[^ ^22^, ^25^ ^]^ Super‐resolution microscopy has overcome the diffraction limit barrier using methods like structured illumination microscopy (SIM)^[^ ^26^ ^]^ and single‐molecule localization microscopy (SMLM),^[^ ^27^, ^28^ ^]^ including STORM^[^ ^27^ ^]^ and PALM,^[^ ^29^ ^]^ which reconstruct high‐resolution images from multiple frames. Yet, the long acquisition times required by these techniques limit their ability to capture rapid or transient nanomotions in living cells. More recent approaches, such as Single‐Frame Super‐Resolution Microscopy (SFSRM),^[^ ^30^ ^]^ improve temporal resolution but still rely on fluorescent labeling and heavy computational processing, which can interfere with live‐cell dynamics and limit real‐time analysis. Thus, a non‐invasive, high‐resolution method capable of capturing fast, subcellular mechanical activity in live cells remains an unmet need.

While optical imaging methods focus on spatial resolution, mechanical sensing approaches such as optical tweezers offer a complementary advantage: extreme sensitivity to nanoscale forces and displacements. Optical tweezers can detect movements at the Ångström scale, apply piconewton forces, and operate at microsecond temporal resolution.^[^ ^31^, ^32^ ^]^ In microrheology, they have been used for both passive (Brownian motion‐based) and active (force‐applied) mechanical measurements,^[^ ^33^ ^]^ revealing insights into cellular stiffness, viscoelasticity, and motor protein function.^[^ ^34^, ^35^ ^]^ However, conventional optical tweezers require the embedding or attachment of probe particles to the cells, which may disrupt native mechanics and obscure subtle nanoscale fluctuations.

**Figure 1:**
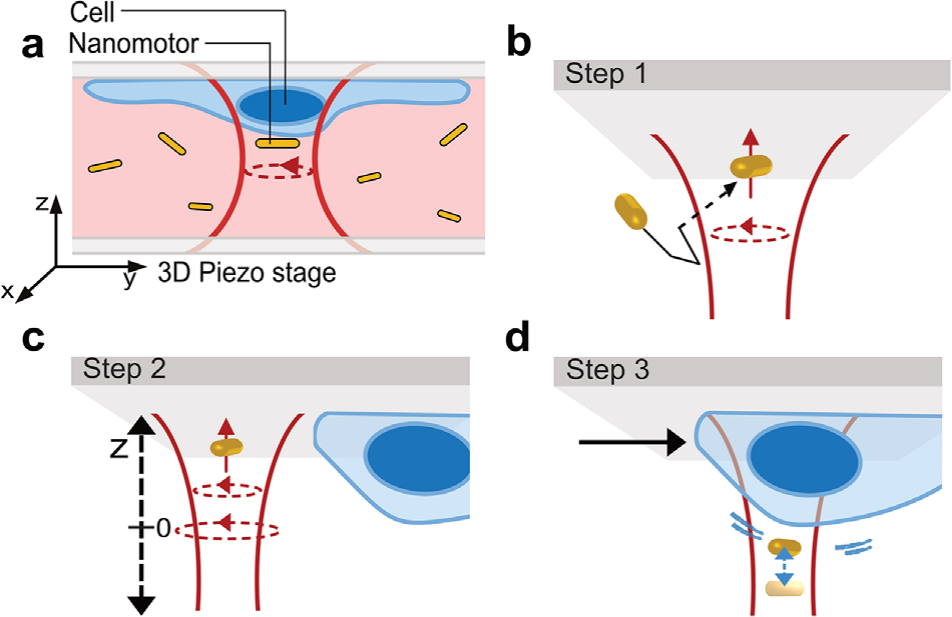
a) Schematic cross‐section of the sample, showing cells adhered to the upper surface and a single nanomotor trapped and rotating above a cell. b–d) Illustration of the measurement procedure: b) Step 1 – Optical trapping of a nanomotor. c) Step 2 – Acquisition of the calibration curve *f*(*z*) by axially translating the piezostage. d) Step 3 – Measurement of rotation frequency fluctuations *f*(*t*) at specific cell locations, followed by extraction of nanomotion amplitudes using the calibration curve.

To address these limitations, we introduce a light‐driven gold nanomotor platform for non‐invasive, high‐precision detection of nanoscale height fluctuations in living cells. In this approach, a circularly polarized laser beam traps a gold nanorod in a 2D optical trap and drives its continuous rotation.^[^ ^36^, ^37^ ^]^ Gold nanorods are well suited for this application due to their biocompatibility and localized surface plasmon resonance (LSPR), which enhances light absorption and scattering and ensures stable optical confinement. Under 2D trapping conditions at the interface of glass‐water or cell‐water, the nanorod aligns parallel to the surface,^[^ ^37^, ^38^, ^39^ ^]^ where circularly polarized light transfers angular momentum and induces high‐speed rotation at kilohertz frequencies along its short axis.^[^ ^36^, ^37^ ^]^ This rotation effectively transforms the nanorod into a nanoscale sensor that transduces cellular activity into a measurable optical signal (**Figure** **1**a). As illustrated in Figure 1b–d, vertical displacements of the cell membrane modulate the nanorod's axial position, which in turn alters its rotation frequency, enabling real‐time readout of nanomotion amplitudes.

By eliminating the need for intracellular probe particles, our method offers an unobstructed view of live‐cell nanomotions. To demonstrate the utility of this approach, we investigate membrane height fluctuations in the HMEC‐1 cells (Human Microvascular Endothelial Cells‐1),^[^ ^40^ ^]^ an immortalized cell line commonly used in studies of cardiovascular disease^[^ ^41^ ^]^ and inflammatory responses.^[^ ^42^ ^]^ We focus on distinct cellular regions, including the nucleus and perinuclear area, to characterize their mechanical behavior with nanometer precision. In contrast to traditional whole‐cell techniques and diffraction‐limited imaging, our approach enables direct, local quantification of localized nanoscale displacements and frequencies. By capturing rapid and transient mechanical events, it provides a powerful new tool for probing cellular mechanics under both physiological and pathological conditions.

## 2 Results and Discussion

### 2.1 Characterization and Validation of the Nanomotor‐Based Detection Platform

**Figure 2:**
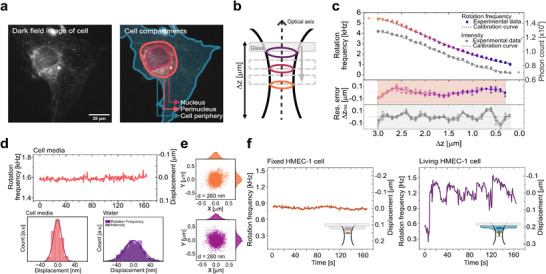
Nanomotor trapping, calibration, and precision of nanomotion detection a) A dark‐field image of an HMEC‐1 cell (left) and schematics of its cellular compartments (right). b) Schematic of calibration curve acquisition via stage displacement. c) Rotation frequency (circles) and scattering intensity (squares) as a function of axial position for a nanomotor (≈ 164 × 98 nm) at 5.5 mW laser power in water. Dashed line: polynomial fit used to generate calibration curves. d) Top: Rotation frequency and corresponding axial displacement over time of a nanomotor trapped against a glass surface at fixed stage position in Cell culture media (CCM). Bottom: Histograms of axial fluctuations for the nanomotor in CCM (left, ≈10 nm SD) and water (right, ≈18 nm SD for frequency‐based and ≈22 nm for intensity‐based detection). e) In‐plane positions of a nanomotor at two axial locations 1 μ m apart, recorded in water using a CMOS camera. *d* indicates the diameter of the probed area, defined as twice the standard deviation of a Gaussian fit. f) Rotation frequency and displacement traces for a nanomotor trapped above a fixed (left) and living (right) HMEC‐1 cell, extracted from autocorrelation function (ACF) analysis.

Before applying the nanomotor platform to living cells, we evaluated its trapping performance, biocompatibility, and calibration under controlled conditions. To enable the nanomotion detection, we employed a customized experimental setup (Figure S1, Supporting Information) incorporating an inverted microscope equipped with a 3D nanopositioning piezo stage, optical trapping laser, and dark‐field microscopy (DFM). DFM clearly resolved distinct cellular compartments, with the nucleus appearing as a uniformly scattering region, the perinuclear zone enriched in bright organelles, and the cell periphery exhibiting weaker scattering (**Figure** **2**a). Against this background, gold nanorods (average size 164 × 98 nm) were easily identified as bright scattering points.

The synthesized nanorods were originally stabilized with the cytotoxic surfactant Cetyltrimethylammonium Bromide (CTAB), which was replaced with biocompatible oligo(ethylene glycol) (OEG)‐terminated alkanethiols forming a self‐assembled monolayer (SAM) on the nanorod surface. We compared hydroxyl‐ and carboxyl‐terminated alkanethiols (AT‐EG_4_‐OH and AT‐EG_6_‐COOH, respectively); while the latter exhibited strong adhesion to cell membranes, the OH‐functionalized rods remained suspended in the trap and rotated freely during long‐term contact with cellular surface. Based on these observations, OH‐functionalized gold nanorods were used in all subsequent experiments. Importantly, cell viability assays confirmed that the functionalized nanorods did not affect HMEC‐1 survival, even after overnight exposure at concentrations 100‐fold higher than those used for trapping (Supporting Information, Section S2, Supporting Information), confirming their suitability for live‐cell studies.

To calibrate the mechanical sensitivity of the system, individual nanorods were trapped and rotated against a glass interface. In this 2D trapping configuration, optical forces and boundary conditions aligned their long axes parallel to the surface, where tilting is strongly suppressed.^[^ ^37^, ^38^, ^39^ ^]^ The sensitivity of rotational frequency to axial displacement was quantified by correlating the frequency response against controlled axial translations of the nanopositioning stage (Figure 2b). The frequency reached its maximum near the laser focus along the z‐axis, consistent with the highest optical torque in this region. The operational range extended to ≈3 μm, limited by reduced gradient forces far from the focus and stochastic motion near the laser focus due to local heating.^[^ ^43^ ^]^ Within this range, the relationship between rotation frequency and displacement along the z‐axis (*f(z)*) was well described by a polynomial fit, allowing calibration curves to be established for direct conversion of frequency fluctuations into displacement amplitudes. These calibration curves were highly reproducible across multiple nanorods and repeated scans (Figures S3 and S4, Supporting Information), confirming the robustness of the approach.

From the calibration curves, the average sensitivity of the system was determined to be 0.55 nmHz^−1^ (Section S.3.1, Supporting Information). The sensitivity and dynamic range of the system could be further tuned by varying the nanorod size. Smaller rods (≈135 × 70 nm) displayed two sensitivity regimes (0.12 and 0.50 nm/Hz; Figure S5, Supporting Information), but were more susceptible to stochastic forces, limiting their usable height range to ≈1.2 μm. Therefore, larger rods (≈ 164 × 98 nm) were used for nanomotion detection, as they provided a broader dynamic range suitable for capturing the large‐amplitude nanomotions of eukaryotic cells.

Tracking rotation frequency of nanorod trapped against glass surface produced a very stable rotational‐frequency signal (Figure 2d (top)). The autocorrelation function (ACF) analysis provided an axial precision of ≈10 nm in cell culture medium (CCM) with a temporal resolution of 0.5 s (Figure 2d (bottom); Figure S6, Supporting Information). Here, precision was defined as the standard deviation of the rotation frequency over time, converted into axial displacement via the calibration curve. This precision approaches the scale of membrane undulations and cytoskeletal‐driven fluctuations (5–50 nm) and is far below the ≈200–300 nm diffraction limit of optical microscopy, making it well suited for monitoring sustained, low‐frequency nanomotions.

For faster dynamic processes, short‐time Fourier transform (STFT) analysis enabled millisecond temporal resolution (Figure S7a, Supporting Information). However, this gain in speed came at the expense of spatial precision (≈130 nm), reflecting the larger frequency uncertainty associated with shorter analysis windows. Despite these differences, the calibration curves derived from ACF and STFT were in excellent agreement (Figure S7d, Supporting Information), confirming the robustness of the frequency‐based readout across timescales.

The measurement environment also had a pronounced effect on performance. In water, the rotational‐frequency signal remained stable but exhibited reduced precision (18 nm vs 10 nm in CCM, as determined by the ACF analysis; Figure 2d (bottom)). This reduction likely arises from the lower viscosity of water, which permits greater stochastic motion of the nanorod, and from the absence of ionic screening, which increases nanorod–surface separation and enhances Brownian perturbations.^[^ ^44^ ^]^ As a result, signal fluctuations were more pronounced, defining the lower sensitivity limit of the system.

Lateral tracking further showed that each nanorod sampled a region of approximately ≈300 nm in diameter (*d*). This sampling area remained nearly constant across the entire axial range, as illustrated in Figure 2c, where measurements taken 1 μm apart along the z‐axis yielded lateral diameters of 260 and 280 nm, respectively. This corresponds to roughly 0.1% of a typical 100 μ m^2^ cell area, underscoring the highly localized, sub‐diffraction character of the readout.

After establishing the calibration and precision of the rotational‐frequency method, we next evaluated whether simpler scattering‐intensity tracking could substitute for rotation frequency‐based readout. Applying both approaches to the same dataset yielded comparable calibration curves, operating ranges (Figure 2c), and axial precision in water (22 nm for intensity‐based and 18 nm for rotation frequency–based detection; Figure 2d, bottom). To assess calibration accuracy, the axial position obtained from the calibration curve (*z*_*cal*_) was compared with the true position set by the piezo stage (*z*_*stage*_). Their difference between those values defines the axial residual error (Δ*z*_*res*_ = *z*_*cal*_ − *z*_*stage*_, see Section S3.4, Supporting Information for more details). The intensity‐based calibration exhibited oscillatory residuals across axial positions (Figure 2c, bottom), most likely caused by interference at the glass interface. In contrast, the rotational‐frequency readout produced smoother and more uniform residuals. Consequently, while scattering‐intensity tracking is a practical alternative for small displacements, its accuracy decreases for larger amplitudes (>100 nm), making the rotation frequency–based approach a more robust and quantitative method for nanomotion detection.

Beyond measurement performance, the physical characteristics of the optical trap itself determine its suitability for live‐cell applications. In particular, plasmonic nanorods enable stable trapping at low near‐infrared powers (5.5 mW), with the 785 nm wavelength lying within the biological transparency window to minimize absorption and scattering artifacts.^[^ ^45^ ^]^ Rotation is driven by the deterministic torque of circularly polarized light, whereas background scattering and Brownian motion introduce only stochastic broadband noise and cannot generate a coherent rotational signature. Moreover, the optical forces exerted by nanomotors are very small (≈0.5 pN^[^ ^44^ ^]^), well below those known to perturb cell behavior, such as the forces 3– to 5 pN generated by cytoskeletal motors^[^ ^46^ ^]^ or the >20 pN applied by AFM tips.^[^ ^47^ ^]^

Because plasmonic nanoparticles inevitably generate localized heating, we evaluated thermal effects using Brownian motion analysis. Localized heating was estimated ≈30 °C at the nanorod surface (Figure S9, Supporting Information), but this excess temperature decays steeply with distance (approximately as 1/*r*), dropping well below surface values within ≈100 nm.^[^ ^37^, ^48^ ^]^ Lateral diffusion of the nanorod further averages the hot spot over ≈300 × 300 nm, preventing prolonged exposure of any single membrane site. Under these conditions, no cell damage or detachment was observed. Previous studies suggest that even such mild, transient heating has been reported to modulate membrane properties, for example by enhancing lipid fluidity or triggering Ca^2 +^ transients,^[^ ^49^, ^50^, ^51^ ^]^ indicating that thermal effects may provide a complementary indicator of cellular activity, rather than limitation.

To further assess the robustness of the optical platform under conditions resembling the irregular topology of cellular membranes, we tested nanomotor performance under spatial confinement. When the optical trap was partially obstructed by a 250‐nm‐tall PDMS structure (Figure S10, Supporting Information), rotational frequency remained stable as long as at least half of the trapping area was accessible. A measurable reduction occurred only under severe confinement, when nanorods were displaced laterally by more than ≈250 nm from the trap center. These results indicate that rotational dynamics are largely insensitive to moderate topographical inhomogeneities, underscoring the suitability of the platform for probing nanoscale mechanics in complex cellular environments.

### 2.2 Nanomotion patterns of HMEC‐1 cells

To detect and quantify cellular nanomotions in HMEC‐1 cells, we developed a three‐step experimental protocol (Figure 1b–d). First, a nanomotor was optically trapped and rotated against a glass surface using a focused laser beam. In the second step, a calibration curve, *f*(*z*), was generated by translating the nanorod axially via the nanopositioning stage and recording its rotation frequency at each height. After calibration, the nanomotor was positioned at the midpoint of the calibrated range to maximize the displacement capacity. In the third step, the nanomotor was laterally relocated to a targeted region on a living cell, where its rotational frequency was continuously monitored over time. Using the *f*(*z*) calibration curve, frequency traces were converted into axial displacement trajectories *z(t)*, enabling precise quantification of cellular nanomotions.

Figure 2d,f shows a typical *z(t)* of a nanomotor trapped against both a glass surface and a living cell. While the glass interface yielded minimal fluctuations, the cellular surface produced large, dynamic displacements. Control measurements performed on fixed HMEC‐1 cells revealed no detectable motion, consistent with the inert glass signal (Figure 2f (left) and Figure S11, Supporting Information). These results confirm the system's high sensitivity to active cellular dynamics and its capability to distinguish living from non‐living mechanical behavior.

**Figure 3:**
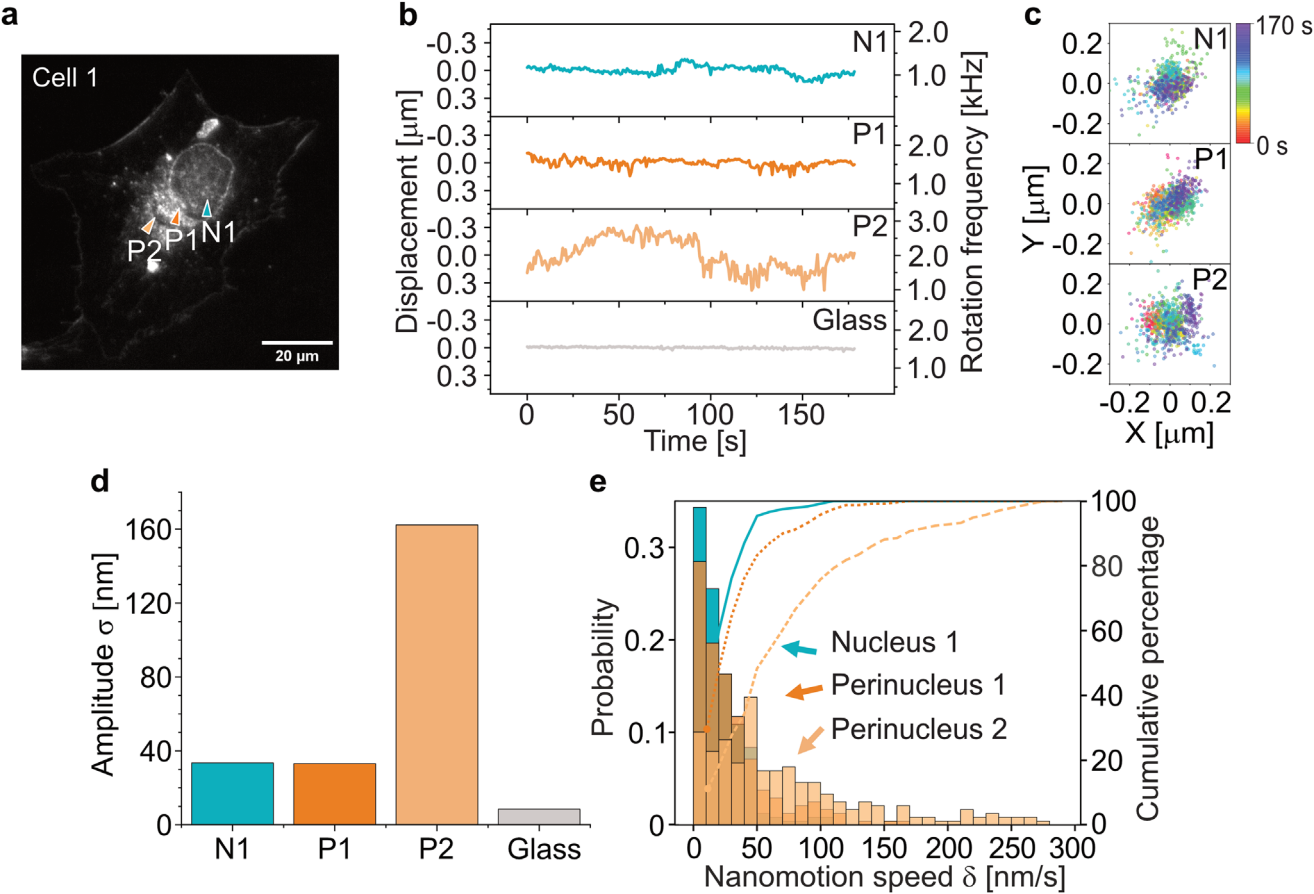
Spatial variability of nanomotion amplitude and speed in a single endothelial cell. a) Dark‐field image of an endothelial cell, with measurement positions indicated by arrows: Nucleus (N1) and two perinuclear locations (P1 and P2). P1 is located closer to the nucleus, while P2 is slightly farther away. b) Nanomotor displacement traces at the positions in (a), along with a reference trace from a glass surface. Nanomotor rotation frequency was determined using the ACF at a sampling rate of ≈1.0 Hz. c) In‐plane position of the nanomotor during measurements at N1, P1, and P2. Recordings consist of 1000 frames at 5 fps, with color indicating nanomotor's position over time. d) Average amplitude σ of nanomotion fluctuations at the three cellular locations, with measurement on glass shown as a reference. e) Probability distribution of nanomotion speeds (nm/s) at the three cellular locations. Measurements were performed sequentially in randomized order. Differences between points, such as the higher activity at P2, reflect local variations in cellular activity.

To explore spatial heterogeneity in cellular mechanics, we measured nanomotion at specific locations in HMEC‐1 cells: the nucleus, perinuclear region, and cell periphery (Figure 2a). A DFM image of a representative HMEC‐1 cell (**Figure** **3**a) shows three probed sites: one over the nucleus (N1) and two in the perinuclear region (P1 and P2). Displacement traces *z(t)* (Figure 3b) revealed large location‐dependent variation and displacement amplitudes locally reaching several hundred nanometers. Of the three sites, P2, the outer perinuclear location, displayed the most pronounced nanomotion activity.

To better understand the local mechanical environment, we simultaneously tracked the nanorod's lateral position during each measurement (Figure 3c). In‐plane fluctuations were observed at all three locations, with particularly large lateral displacements occurring at P2 (see Videos [Link], [Link], [Link], Supporting Information). Yet, the nanomotor remained stably confined within the optical trap. As previously demonstrated, lateral displacements within the ≈300 ×300 nm trapping area have minimal impact on rotational frequency. The combination of substantial axial and lateral displacements at P2 thus confirms the elevated mechanical activity in this region.

To quantify nanomotion amplitude, we calculated the standard deviation σ of the displacement trace *z*(*t*), defined as $\sigma =\sqrt{\left\langle {\left(z\left(t\right)-\bar{z}\right)}^{2}\right\rangle }$.^[^ ^19^, ^52^ ^]^ The nuclear (N1) and perinuclear (P1) sites exhibited similar amplitude values of 33.4 and 33.1 nm, respectively (Figure 3d). In contrast, the outer perinuclear site P2 displayed a markedly higher amplitude of 162 nm. As a reference, nanomotor fluctuations measured on a glass surface were substantially lower, with σ = 9 nm, highlighting the contrast between live‐cell and inert environments.

To further characterize the dynamics, we calculated the instantaneous nanomotion speed as the vertical displacement between consecutive 0.67‐second intervals (Figure 3e). Motion at the nuclear site (N1) was relatively slow, with 90% of displacements occurring below 50 nm s^−1^. The perinuclear site P1 showed similar behavior, with 85% of speeds below this threshold. However, at P2, only 55% of motion fell below 50 nm s^−1^, and 10% exceeded 200 nm s^−1^, showing significantly more active dynamics. Average speeds followed a similar trend: ${\bar{\delta }}_{N1}=20$ nm s^−1^, ${\bar{\delta }}_{P1}=30$ nm s^−1^, and ${\bar{\delta }}_{P2}=76$ nm s^−1^. These differences underscore the spatial variability in mechanical activity across subcellular regions. Additional examples from Cells 2 and 3 are presented in Figure S12 (Supporting Information), corresponding to the cell images shown in Figure 5a,b.

**Figure 4:**
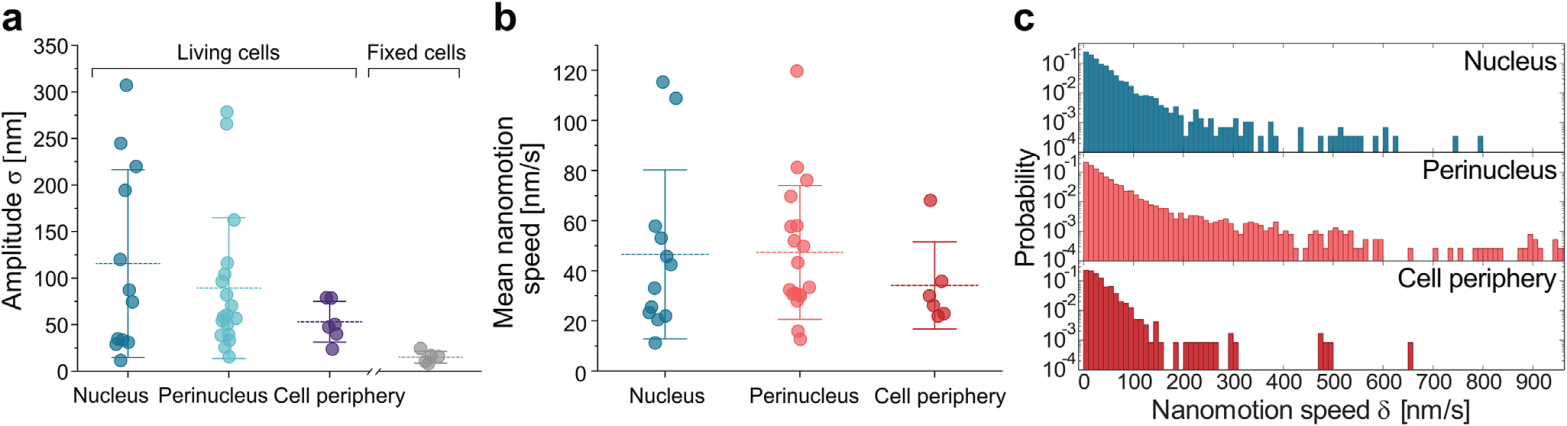
Compiled nanomotion amplitude and speed across cellular regions. Data from 37 living cell measurements, categorized by cellular region as defined in Figure 2a, reveal spatial variability in nanomotion dynamics. a) Average nanomotion amplitude (σ) from living cells, together with fixed‐cell controls. Fixed‐cell amplitudes matched those on bare glass, confirming the absence of nanomotion. b) The corresponding average nanomotion speed (δ). c) Probability distribution of nanomotion speeds on a logarithmic scale. Measurements were conducted sequentially in randomized order. Each trace was recorded for 180s and analyzed using ACF at an effective sampling rate of 1 Hz. Sample sizes: nucleus (*n* = 12), perinucleus (*n* = 19), cell periphery (*n* = 6), and fixed cells (*n* = 6).

**Figure 5:**
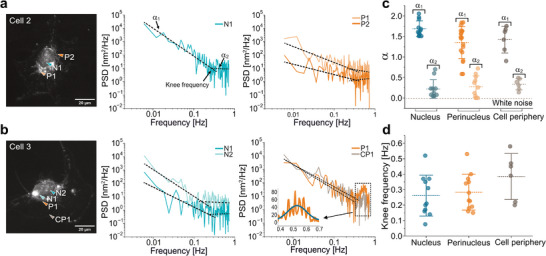
Nanomotion frequency and power spectral density analysis. a,b) Nanomotion frequency analysis in two endothelial cells. Left: DFM images of the cells, with arrows indicating the nanomotion measurement locations using a single nanomotor. Middle and Right: Power Spectral Density (PSD) plots of the motion amplitude recorded over three minutes. The 1/f^α^ fits are represented with a dashed line, where the α_1_ is the fitting parameter for the low‐frequency region, and α_2_ corresponds to the high‐frequency region. The knee frequency is defined as the intersection point of the two 1/f^α^ fits. In the PSD spectrum of Cell 3 (right), the inset shows a peak corresponding to oscillations, fitted with a Gaussian centered at 0.53 ± 0.14 Hz. c,d) Values of α_1_ and α_2_ (c) and knee frequencies (d) obtained by fitting the PSD spectra from 37 nanomotion measurements. Data are categorized by cellular region: nucleus (*n* = 12), perinucleus (*n* = 19), and cell periphery (*n* = 6). Analysis was made with ACF.

To uncover broader patterns in subcellular mechanical activity, we compiled 37 measurements from 16 cells, comparing the average nanomotion amplitude (σ), average speed (δ), and instantaneous speed across the nucleus, perinuclear region, and cell periphery (**Figure** **4**). For reference, Figure 4a also includes fixed‐cell controls, which yielded amplitudes comparable to bare glass, confirming the absence of detectable nanomotion. Measurements on living cells were concentrated in the nuclear and perinuclear regions, which are hubs of mechanical and biochemical activity,^[^ ^53^ ^]^ with fewer taken at the comparatively stable periphery. The nucleus exhibited the largest nanomotion amplitudes (${\bar{\sigma }}_{N}$ = 115.6 ± 101 nm), but relatively moderate speeds. This combination suggests slow but large‐scale fluctuations, consistent with nuclear processes such as chromatin remodeling, gene transcription, and nuclear envelope dynamics.^[^ ^54^ ^]^ In contrast, the perinuclear region showed slightly lower amplitudes (${\bar{\sigma }}_{P}$ = 89.3 ± 75 nm) but higher speeds (${\bar{\delta }}_{P}$ = 48 ± 27 nm s^−1^), with more frequent fast movements (Figure 4c). These frequent and fast movements likely reflect the cytoskeletal rearrangements in this region, as well as the endoplasmic reticulum and Golgi trafficking activity.^[^ ^55^ ^]^ The cell periphery emerged as the most mechanically stable region. It showed the lowest amplitude (${\bar{\sigma }}_{CP}$ = 53.1 ± 22 nm) and speed (${\bar{\delta }}_{CP}$ = 34 ± 17 nm s^−1^) with instantaneous speeds typically below 100 nm s^−1^. These relatively smaller nanomotions are consistent with the structural role of the cell edge in adhesion and shape maintenance,^[^ ^56^ ^]^ and likely reflect local factors, such as fewer organelles and reduced trafficking.

### 2.3 Frequency‐Resolved Mechanics of Cellular Nanomotion

**Figure 6:**
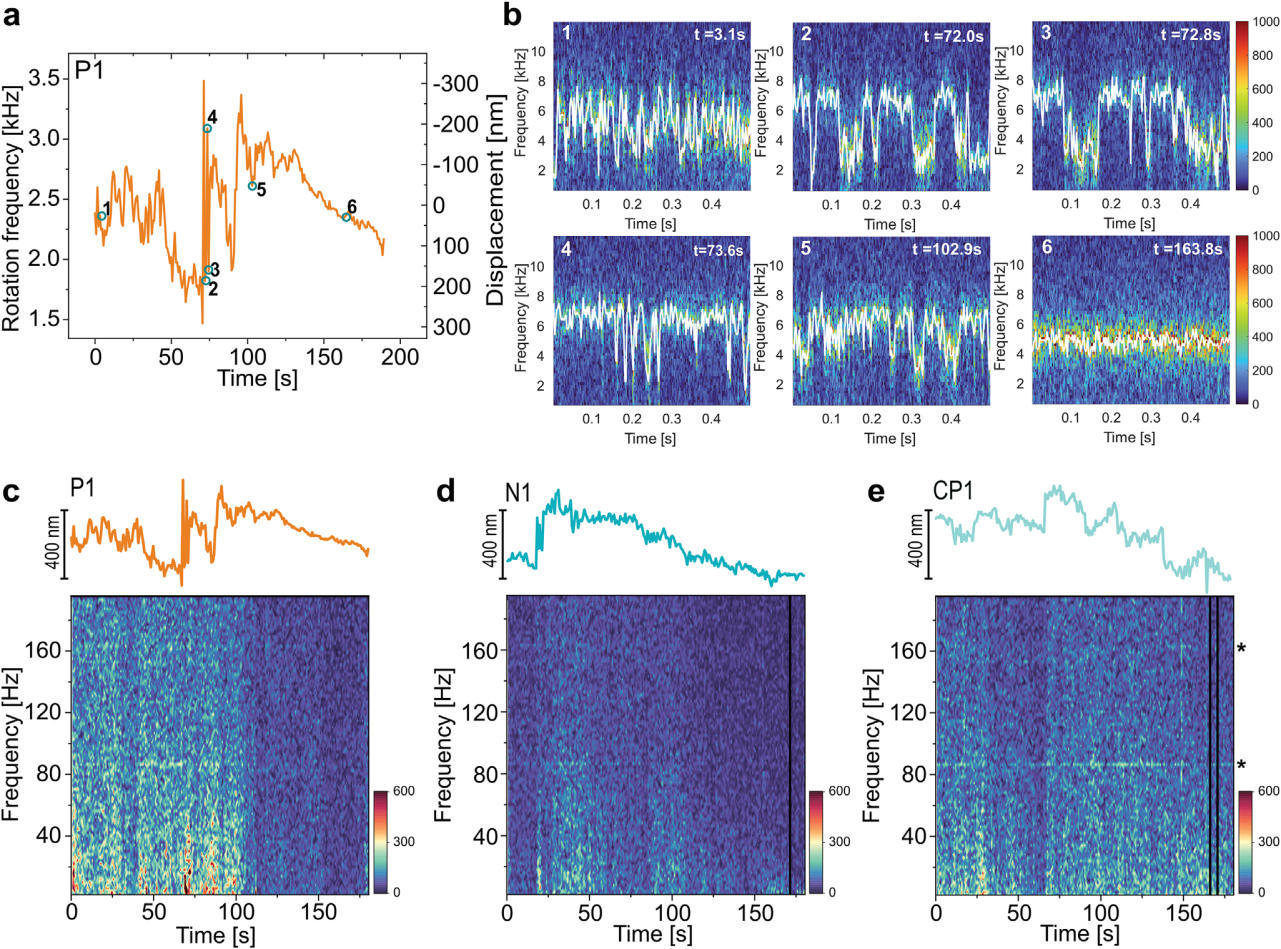
Exploratory analysis of transient and oscillatory nanomotions. a) Nanomotion trace recorded from the perinuclear region of Cell 3 obtained using ACF analysis. b) STFT spectrograms of the backscattered light intensity at selected time points from (a), showing peak at 2*f*. The white line highlights fluctuations in the rotation frequency over time. Together, these results illustrate how the nanomotor platform can capture both stable and transient nanomotions across subcellular regions. c–e) Top: Nanomotion traces measured at three distinct locations on Cell 3, as shown in Figure 5b (nucleus, perinuclear, and periphery). Bottom: Corresponding Fourier spectrograms of *f(t)* extracted using STFT. No persistent periodic frequencies were detected in the spectrograms. Black asterisks in (e) indicate frequency peaks originating from environmental interference. STFT was computed using 512‐sample Hanning windows (50% overlap), corresponding to a 5.12 ms analysis window.

To gain a deeper insight into the temporal characteristics of cellular nanomotions, we analyzed the frequency components of axial displacement traces *z*(*t*), by computing their power spectral density (PSD). The PSD provides a frequency‐domain representation of motion dynamics, revealing dominant periodicities and the underlying temporal correlations. Representative PSD spectra from two cells (Cell 2 and Cell 3) are shown in **Figures** **5**a,b,**6**e, alongside DFM images indicating measurement locations in the nuclear (N), perinuclear (P) and cell periphery (CP) regions. Complete PSD data from all cells are provided in the Figures S.13–S.15 (Supporting Information). As observed previously, nanomotion amplitudes varied considerably between spatially adjacent regions within the individual cells (e.g., N1 and P1 in both cells) and even among multiple sites within the same region (e.g., P1 and P2 in Cell 2, and N1 and N2 in Cell 3). Despite this spatial variability, the PSD profiles across all measurements consistently followed a power‐law decay with frequency approximated by a 1/f^α^ frequency pattern (Figure 5a,b middle and right panels). This scale‐invariant behavior revealed two distinct regimes, each characterized by a different scaling exponent (α). In the low‐frequency regime (< 0.1 Hz) the PSD showed a steep decline, with higher scaling exponent (α_1_ ≈ 0.7–2), indicating pronounced long‐range correlations in the nanomotion signals. Such behavior is commonly observed in complex biological systems exhibiting memory effects, including heart rate variability, neural dynamics, and bacterial motion.^[^ ^19^, ^52^, ^57^ ^]^ Conversely, in the high‐frequency regime (> 0.1 Hz) the PSD became flatter, with lower scaling exponents (α_2_ ≈ 0.2), suggesting reduced correlation and more stochastic behavior at shorter timescales. Figure 5c summarizes the extracted α_1_ and α_2_ values for all measurements, grouped by cellular region.

Distinct trends emerged when comparing low‐frequency exponents across regions. The nucleus consistently showed the highest degree of temporal correlation, with an average exponent of ${\bar{\alpha }}_{N}^{1}=1.69\pm 0.2$, approaching values typical of processes governed by long‐memory dynamics. This behavior may reflect the mechanical and biochemical environment of the nucleus, including chromatin remodeling, transcriptional activity, and the viscoelastic response of the nuclear lamina.^[^ ^58^ ^]^ In contrast, the perinuclear (${\bar{\alpha }}_{P}^{1}=1.35\pm 0.4$) and peripheral (${\bar{\alpha }}_{CP}^{1}=1.42\pm 0.3$) regions displayed broader distributions of ${\bar{\alpha }}^{1}$ ranging from ≈0.5 to 1.7. This diversity likely arises from heterogeneous and dynamic processes in the cytoplasm.^[^ ^59^ ^]^

To better characterize the transition between these two regimes, we extracted the “knee frequency” *f* ^*K*^, the frequency at which the PSD slope changes, marking a shift in the dominant mechanical behavior.^[^ ^60^ ^]^ Remarkably, the average *f* ^*K*^ was similar across all regions:$f_{N}^{K}=0.26\pm 0.13$ Hz for the nucleus, $f_{P}^{K}=0.28\pm 0.11$ Hz for the perinucleus, and $f_{CP}^{K}=0.39\pm 0.15$ Hz for the cell periphery (Figure 5d). This consistency suggests that the mechanical transition between correlated and stochastic regimes is a general feature of cellular nanomotion, rather than one restricted to a specific subcellular structure. Previous studies have observed similar spectral transitions under externally applied forces across various time and length scales.^[^ ^61^, ^62^ ^]^ Our results indicate that comparable transitions also occur due to intrinsic cellular activity.

### 2.4 Detection of Transient and Oscillatory Nanomotions

In addition to the broad nanomotion spectra described above, the platform also revealed short‐lived oscillatory components in specific regions. Although most PSD plots showed broad distributions without sharp peaks, we occasionally observed distinct oscillatory components, suggesting the presence of periodic processes in specific regions. For example, in the perinuclear region of Cell 3 (Figure 5), PSD revealed a clear peak at 0.53 ± 0.14 Hz. Figure 6a shows the corresponding displacement trace *z(t)* derived using the ACF with 0.5‐s time frames. Between approximately 70 and 76 s, we observed transient oscillations reaching amplitudes of up to 600 nm.

To resolve these rapid fluctuations in greater detail, we applied the STFT analysis to the same measurement (Figure 6b). During the first 50 s, the ACF data showed high‐amplitude fluctuations, matched by rapid changes in STFT‐derived frequencies on the millisecond scale. At 70.4 s, the nanomotor's rotation frequency abruptly increased by 1 kHz, corresponding to a vertical displacement of ≈500 nm, followed by a burst of height oscillations lasting until ≈76 s. During this interval, the rotation frequency alternated between two discrete states, at rate exceeding 10 Hz. After this active phase, the signal gradually diminished, and by ≈120 s, the nanomotor resumed stable rotation, suggesting a return to slower, more gradual cellular changes. These dynamics highlight the transient nature of cellular nanomotions, which can shift rapidly between quiescent and active states within seconds.

Interestingly, similar oscillatory behavior was observed in the nuclear region of the same cell. As shown in Figure 6d and Figure S16 (Supporting Information), around 20 s into the measurement, the nucleus exhibited semi‐regular oscillations with amplitudes of 150–200 nm and a frequency of ≈10 Hz. This bi‐state pattern, also detected via STFT, further supports the notion that such oscillatory nanomotions are a widespread but transient phenomenon. These types of nanoscale mechanical oscillations, with amplitudes and frequencies in the 10 Hz range, are rarely reported, likely due to their brief duration and localized origin, which are challenging to capture using conventional techniques. However, similar events have been observed in the peripheral endoplasmic reticulum of fibroblasts and endothelial cells by Nixon‐Abell et al.,^[^ ^63^ ^]^ with dynamics closely matching our findings.

To further explore the frequency characteristics of cellular nanomotions, we performed Fourier analysis on the displacement signals *z(t)* obtained using the STFT method. This allowed us to investigate nanomotion frequencies in the 1–200 Hz range across three locations on Cell 3 (Figure 6c–e). While no persistent periodicities were observed within this frequency band, we occasionally detected transient peaks around 86 and 163 Hz (indicated by asterisks on Figure 6e). However, these peaks were also present in control measurements on glass (Figure S18, Supporting Information), confirming that they originated from environmental noise rather than biological activity. Despite the absence of sustained periodic signals, several trends emerged. In the perinuclear region (Figure 6c), nanomotion activity spanned the full 1–200 Hz range and remained high until approximately 120 s, after which it declined markedly across all frequencies. A similar temporal pattern was observed at the cell periphery, with broad spectral activity and a prominent band centered around 20 Hz. Across all regions, large‐amplitude displacements were closely associated with increased spectral power in the 1–40 Hz range (Figure 6c–e). These fluctuations are consistent with prior reports attributing mechanical activity in this range to the collective dynamics of molecular motors and the cytoskeleton.^[^ ^64^ ^]^ In contrast, the nucleus exhibited lower overall spectral activity, with nanomotion concentrated in the 1–20 Hz range. This activity appeared in discrete bursts, particularly between 25–50 s and 80–120 s (Figure 6d). These nuclear bursts likely correspond to co‐regulated nuclear processes, such as transcription, DNA replication, and repair.^[^ ^59^ ^]^

Together, these exploratory findings highlight the capacity of optically driven nanomotors to resolve transient, frequency‐dependent mechanical dynamics across distinct subcellular compartments. By capturing both steady‐state and transient spatiotemporal patterns, the nanomotor platform provides complementary insights into the localized mechanical behavior of living cells. While the observed frequency fluctuations primarily reflect axial nanomotion, other membrane processes could in principle contribute. Local variations in protein density or lipid organization may influence hydrodynamic coupling, though such effects would need to be substantial to be detected given the ≈300 × 300 nm averaging area. Electrostatic contributions are minimized by the SAM coating and further screened under physiological ionic strength.^[^ ^65^ ^]^ Although difficult to isolate in the present dataset, such biochemical–mechanical couplings could provide an additional layer of sensitivity to cellular state and represent an interesting direction for future mechanophenotyping.

Finally, while our study focused on HMEC‐1 endothelial cells, nanoscale mechanical fluctuations have been reported across diverse cell types, indicating that this phenomenon is not restricted to a single lineage. Previous research on yeast^[^ ^66^ ^]^ and bacteria^[^ ^67^ ^]^ has demonstrated the potential of nanoscale mechanical sensing, with bacterial studies even employing machine learning to classify oscillatory behaviors based on virulence status. Similarly, Peng et al.^[^ ^68^ ^]^ tracked nanomechanical vibrations during oocyte development, illustrating the broad applicability of these approaches.

## 3 Conclusion

We developed and validated a nanomotor‐based detection platform capable of resolving nanoscale cellular motions with high spatial and temporal precision. By leveraging the strong optical response of plasmonic nanorods, the method achieves ≈10 nm axial sensitivity with ACF analysis and millisecond temporal resolution with STFT analysis. In addition, it provides high lateral localization precision (≈300 ×300 nm), offering a complementary approach to traditional techniques such as AFM or QCM by enabling both subcellular spatial resolution and high temporal responsiveness.

Light‐driven nanomotors represent a powerful tool for nanoscale mechanobiology. By bridging structural imaging with functional dynamics, this platform enables direct quantification of subcellular mechanical behavior, revealing spatial heterogeneity and transient events often missed by conventional approaches. The results presented here highlight its potential for advancing fundamental cell biology and for driving innovation in mechanophenotyping, diagnostics, and therapeutic development.

Looking ahead, the platform could be extended through integration with fluorescence or super‐resolution microscopy to correlate mechanical fluctuations with molecular features. Functionalizing nanomotors with ligands may enable targeted studies of receptor–ligand interactions or organelle‐specific mechanics, while adaptation to multiplexed formats could allow high‐throughput profiling of pharmacological responses or disease‐associated mechanical signatures at the single‐cell level. Together, these directions underscore the versatility of light‐driven nanomotors as a next‐generation approach for probing cell mechanics and biomedical research and precision diagnostics.

## 4 Experimental Section

### Synthesis and Functionalization of Gold Nanorods

Gold nanorods were synthesized by a seed‐mediated growth protocol.^[^ ^37^ ^]^ Unless otherwise noted, experiments were performed with (164±8)nm × (98±5) nm GNRs, whose longitudinal plasmon resonance overlaps the trapping wavelength; results with smaller rods (140±9)nm × (70±4) nm were indicated explicitly. To enhance biocompatibility and reduce cytotoxicity, the CTAB capping layer was replaced with SAMs of oligo(ethylene glycol) alkanethiols, either hydroxy‐terminated (HS‐(CH_2_)_11_EG_4_OH, abbreviated AT‐EG_4_‐OH) or carboxy‐terminated (HS‐(CH_2_)_11_EG_6_COOH, abbreviated AT‐EG_6_‐COOH) (ProChimia, Poland). Compounds were dissolved in ethanol (10 mM) and mixed with the nanorod solution to a final concentration of 0.5 mM, corresponding to a CTAB:thiol ratio of approximately 1:4.4. The mixture was stored overnight at 4 °C, centrifuged at 7500 rpm to remove the supernatant, and re‐suspended in Milli‐Q water. This washing step was repeated, and the solution stored overnight prior to use. Before cell experiments, 70% of the supernatant was replaced with fresh Milli‐Q water. COOH‐functionalized rods exhibited strong adhesion to cell membranes, preventing continuous rotation, whereas OH‐functionalized rods remained suspended in the trap and rotated freely. Based on these results, OH‐functionalized nanorods were used in all experiments. For measurements on living cells, 1 μL of functionalized nanorod suspension was mixed with 500 μ L of cell medium and applied to the sample.

### Optical Tweezers Setup

The optical‐tweezers setup (Figure S1, Supporting Information) was based on an inverted microscope (Nikon TE300) equipped with a 785nm trapping beam (Toptica Photonics), 60x/0.70 NA objective, 3D nanopositioning piezostage (Nano‐LPMW, Mad City Labs), dark‐field illumination and CMOS camera (Andor, Neo 5.5 sCMOS).

The back‐scattered light from individual trapped nanorods was passed through a linear polarizer and collected with a single‐photon counting photomultiplier tube (PMT; Becker & Hickle). PMT signals were recorded for 0.5 s at a sampling rate of 100 kHz, with a 0.5 s repetition interval, yielding an effective measurement period of 1 s per cycle. In case when total scattering intensity was used as measure of the axial position, the photon counts were integrated over each interval to determine the total scattering intensity. To determine the rotation frequency the autocorrelation was calculated and fitted with autocorrelation function (ACF) using: $C\left(\tau \right)=I_{0}^{2}+0.5I_{1}^{2}\exp\left(-\frac{\tau }{\tau_{0}}\right)\cos\left(4\pi f\tau \right)$, where *I*_0_ is the average intensity, *I*_1_ is amplitude of intensity fluctuation, *f* is the average rotation frequency, and τ_0_ is the autocorrelation decay time. The parameter τ_0_ is closely associated with rotational Brownian motion and is defined by: $\tau_{0}=\frac{\gamma_{r}}{4k_{B}T_{r}}$ where *T*_*r*_ is the rotational Brownian temperature, *k*_*B*_ Boltzmann's constant, and γ_*r*_(*T*) is the friction coefficient given by πη(*T*)*gL* ^3^. Here, η(*T*) represents the temperature‐dependent dynamical viscosity of the surrounding water, *g* represents the geometrical shape factor dependent on the nanorod's eccentricity, and *L* is the length of the nanorod.^[^ ^36^, ^37^ ^]^

For comparison of analysis on scattering intensity with rotation‐based nanomotion detection, scattering intensity was also analyzed directly by integrating the total photon count over 0.5 s, without ACF fitting.

In addition to ACF, STFT analysis was applied to achieve higher temporal resolution. PMT signals were sampled at 100 kHz and processed in 512‐sample Hanning windows with 256‐sample overlap, corresponding to a 5.12 ms window, 2.56 ms step size, and 195 Hz frequency‐bin spacing. STFT spectrograms exhibited a peak at twice the rotation frequency 2*f*. The position of this peak, determined as the location of the local maxima, was tracked over time to obtain the rotation frequency as a function of time (*f*(*t*)) with enhanced temporal resolution. The rotation frequency was converted to displacement profiles *z(t)* using previously established calibration curves *f(z)*. ACF and Fourier‐based analyzes are mathematically related and provide consistent readouts of rotational dynamics. Here, ACF emphasizes low‐frequency fluctuations with high spatial precision, while STFT resolves transient, high‐frequency events. Using both methods ensures complementary perspectives and facilitates comparison with prior optical‐tweezers studies, where both approaches were commonly employed.^[^ ^69^ ^]^ In‐plane lateral resolution was determined by tracking in‐plane motion of nanorods using the CMOS camera under 5.5 mW trapping using DFM at 1400 fps. The diameter of the probed area *d* was defined as twice the standard deviation of a Gaussian fit. Each recording comprised 5000 frames.

### Cell Culture and Sample Preparation

HMEC‐1 cells were cultured in an atmosphere of 5% *CO*_2_ at 37°C in MCDB 131 medium (Gibco, Fisher Scientific, USA) supplemented to a total concentration of 10 mM L‐glutamine, 10 ng mL^−1^ epidermal growth factor (EGF), 1 μg mL^−1^ hydrocortisone, 10% fetal bovine serum (FBS), 1% non‐essential amino acids, and 1% penicillin/streptomycin. Prior to experiments, cells were grown to a confluency of 80‐90% and then harvested using TrypLE Express Enzyme (Gibco, Thermo Fisher Scientific, USA) for 7 min, followed by centrifugation at 200 × g for 7 min. The supernatant was removed and the cell pellet was re‐suspended in fresh media and diluted to a corresponding confluency of ≈30%. Cells were seeded onto cleaned glass cover‐slips by allowing them to sediment sparsely onto the cover‐slips in a culture dish for 2 h. The cover slip with attached HMEC‐1 cells was used as the top layer of the sample chamber. After cell adhesion, the coverslip was gently washed with cell media to remove cellular debris. Cells located at the periphery of the coverslip were removed using a lint‐free tissue to ensure a tight seal of the chamber. A 120 μm spacer was placed on the coverslip, and 3 μL of a GNR solution at an approximate concentration of 10^7^ per mL in the MCDB‐131 medium, was dropcasted onto the cover slip. The measurement chamber was then sealed with a microscope cover glass slide. Measurements were conducted immediately after sample preparation, under conditions that minimize time for cellular uptake of nanorods. No intracellular nanorods were detected during the course of the experiments.

For the control measurements on fixed HMEC‐1 cells, the cells were cultured and seeded on glass coverslips as described above. The fixation steps were performed in room temperature. The cells were washed three times with cold (4°C) phosphate‐buffered saline (PBS), with five‐minute intervals between washes. After washing, the cells were fixed with cold 4% formaldehyde for 15 min. Following fixation, the cells were rinsed three times with PBS and then washed an additional three times, again with five‐minute intervals between washes. Finally, the cells were stored in PBS at (4°C) until measurement.

### Nanomotion Measurements

The sample was mounted on a microscope, and a single nanorod was optically trapped against the coverslip. The microscope stage was adjusted to the position above laser focus where the rotation of the nanorod was the lowest. The laser power was set to the minimum level that allowed stable trapping of the nanorod over an axial range of several micrometers (typically 4–5.5 mW). Calibration was performed by lowering the stage in 100 nm steps, recording five intensity traces at each position, and fitting the averaged intensity from these measurements with a second‐order polynomial to generate the calibration curve *f*(*z*). After calibration, the nanorod was positioned above regions of interest, and data were recorded for 240 measurement cycles, yielding a 180 s trace. Measurements were repeated at multiple sites in random order, with no evidence of order‐dependent decline in cellular activity.

In this experiments, the trapping wavelength (785 nm) falls within the biological near‐infrared window, where cellular absorption and scattering were minimal, thereby reducing potential optical artifacts.^[^ ^45^ ^]^ Nanorod rotation was driven by the deterministic torque of circularly polarized light, while background scattering and Brownian motion contribute only stochastic, broadband noise that cannot generate or modulate a coherent rotational signature. The absence of detectable signals in fixed‐cell controls further confirms that the rotation frequency fluctuations observed in living cells originate from dynamic biological activity rather than from optical scattering artifacts or thermal noise.

### Statistical Analysis

No further preprocessing of the raw PMT or video signals was performed. Displacement amplitudes were quantified as the standard deviation (σ) of each trace and are reported as mean ± standard deviation (SD), with sample sizes (n) specified in figure captions. All nanomotion measurements on living cells were treated individually and compared against glass controls. Group differences were assessed by one‐way analysis of variance (ANOVA) with a significance level of p = 0.05. For living‐cell measurements, all regions showed significantly higher nanomotion amplitudes than glass. As an additional control, nanomotions of fixed cells were measured with a single nanomotor across three subcellular positions. One‐way ANOVA indicated no significant differences between fixed‐cell and glass measurements, nor among positions on the fixed cell. For cell viability assays, viability was calculated as the percentage of live cells relative to total nuclei. Two independent experiments (*n* = 2) were performed, and results are presented as mean ± SD. Statistical analysis and plotting were performed in OriginPro 2023 (OriginLab) and ImageJ (NIH) for image‐based quantification.

## Conflict of Interest

The authors declare no conflict of interest.

## Supporting information

Supporting Information

Supplemental Video 1

Supplemental Video 2

Supplemental Video 3

## Data Availability

The data that support the findings of this study are available from the corresponding author upon reasonable request.

## References

[1] J. Kim , Sci. Rep. 2020, 10, 2301. 32041981 10.1038/s41598-020-59030-2 PMC7010710

[2] E. Schäfer , M. Tarantola , E. Polo , C. Westendorf , N. Oikawa , E. Bodenschatz , B. Geil , A. Janshoff , PLoS One 2013, 8, e54172. 23349816 10.1371/journal.pone.0054172 PMC3547869

[3] G. Peyret , R. Mueller , J. d'Alessandro , S. Begnaud , P. Marcq , R.‐M. Mège , J. M. Yeomans , A. Doostmohammadi , B. Ladoux , Biophys. J. 2019, 117, 464. 31307676 10.1016/j.bpj.2019.06.013 PMC6697349

[4] F. E. Nolet , A. Vandervelde , A. Vanderbeke , L. Piñeros , J. B. Chang , L. Gelens , eLife 2020, 9, e52868. 32452767 10.7554/eLife.52868 PMC7314552

[5] M. Mojica‐Benavides , D. D. van Niekerk , M. Mijalkov , J. L. Snoep , B. Mehlig , G. Volpe , M. Goksör , C. B. Adiels , Proc. Natl. Acad. Sci. USA 2021, 118, 6. 10.1073/pnas.2010075118 PMC8017953 33526662

[6] S. Flemming , F. Font , S. Alonso , C. Beta , Proc. Natl. Acad. Sci. USA 2020, 117, 6330. 32161132 10.1073/pnas.1912428117 PMC7104017

[7] N. Inagaki , H. Katsuno , Trends Cell Biol. 2017, 27, 515. 28283221 10.1016/j.tcb.2017.02.003

[8] S. Suresh , Acta Biomater. 2007, 3, 413. 17540628 10.1016/j.actbio.2007.04.002 PMC2917191

[9] S. Wu , X. Liu , X. Zhou , X. M. Liang , D. Gao , H. Liu , G. Zhao , Q. Zhang , X. Wu , Biosens. Bioelectron. 2016, 77, 164. 26406457 10.1016/j.bios.2015.09.024

[10] S. Kasas , F. S. Ruggeri , C. Benadiba , C. Maillard , P. Stupar , H. Tournu , G. Dietler , G. Longo , Proc. Natl. Acad. Sci. USA 2015, 112, 378. 25548177 10.1073/pnas.1415348112 PMC4299216

[11] A. C. Kohler , L. Venturelli , G. Longo , G. Dietler , S. Kasas , The Cell Surface 2019, 5, 100021. 32743137 10.1016/j.tcsw.2019.100021 PMC7388971

[12] A. Mustazzolu , L. Venturelli , S. Dinarelli , K. Brown , R. A. Floto , G. Dietler , L. Fattorini , S. Kasas , M. Girasole , G. Longo , Antimicrob. Agents Chemother. 2019, 63, e02194. 30602518 10.1128/AAC.02194-18 PMC6395931

[13] M. Tarantola , A.‐K. Marel , E. Sunnick , H. Adam , J. Wegener , A. Janshoff , Integr. Biol. 2010, 2, 139. 10.1039/b920815a 20473392

[14] K. Syal , R. Iriya , Y. Yang , H. Yu , S. Wang , S. E. Haydel , H.‐Y. Chen , N. Tao , ACS Nano 2016, 10, 845. 26637243 10.1021/acsnano.5b05944

[15] C.‐T. Yang , R. Méjard , H. J. Griesser , P. O. Bagnaninchi , B. Thierry , Anal. Chem. 2015, 87, 1456. 25495915 10.1021/ac5031978

[16] F. Cavallini , M. Tarantola , Prog. Biophys. Mol. Biol. 2019, 144, 116. 30025825 10.1016/j.pbiomolbio.2018.06.010

[17] T.‐H. Tung , S.‐H. Wang , C.‐C. Huang , T.‐Y. Su , C.‐M. Lo , Sensors 2020, 20, 3250.32517325

[18] D. Opp , B. Wafula , J. Lim , E. Huang , J.‐C. Lo , C.‐M. Lo , Biosens. Bioelectron. 2009, 24, 2625. 19230649 10.1016/j.bios.2009.01.015 PMC2668605

[19] I. E. Rosłoń , A. Japaridze , P. G. Steeneken , C. Dekker , F. Alijani , Nat. Nanotechnol. 2022, 17, 637. 35437320 10.1038/s41565-022-01111-6

[20] J. Mertens , A. Cuervo , J. L. Carrascosa , Nanoscale 2019, 11, 17689. 31538998 10.1039/c9nr05240b

[21] G. Longo , L. Alonso‐Sarduy , L. M. Rio , A. Bizzini , A. Trampuz , J. Notz , G. Dietler , S. Kasas , Nat. Nanotechnol. 2013, 8, 522. 23812189 10.1038/nnano.2013.120

[22] G. Popescu , Y. Park , N. Lue , C. Best‐Popescu , L. Deflores , R. R. Dasari , M. S. Feld , K. Badizadegan , Am. J. Physiol.: Cell Physiol. 2008, 295, C538. 18562484 10.1152/ajpcell.00121.2008 PMC2518415

[23] C. Cordeiro , O. J. Abilez , G. Goetz , T. Gupta , Y. Zhuge , O. Solgaard , D. Palanker , Biomed. Opt. Express 2017, 8, 4652. 29082092 10.1364/BOE.8.004652 PMC5654807

[24] Z. Guo , C.‐T. Yang , C.‐C. Chien , L. A. Selth , P. O. Bagnaninchi , B. Thierry , Small Methods 2022, 6, 2200471. 10.1002/smtd.202200471 35764869

[25] P. Y. Liu , L. K. Chin , W. Ser , H. F. Chen , C.‐M. Hsieh , C.‐H. Lee , K.‐B. Sung , T. C. Ayi , P. H. Yap , B. Liedberg , K. Wang , T. Bourouina , Y. Leprince‐Wang , Lab Chip 2016, 16, 634. 26732872 10.1039/c5lc01445j

[26] M. G. Gustafsson , J. Microsc. 2000, 198, 82. 10810003 10.1046/j.1365-2818.2000.00710.x

[27] M. J. Rust , M. Bates , X. Zhuang , Nat. Methods 2006, 3, 793. 16896339 10.1038/nmeth929 PMC2700296

[28] A. Sharonov , R. M. Hochstrasser , Proc. Natl. Acad. Sci. USA of the United States of America 2006, 103, 18911. 10.1073/pnas.0609643104 PMC1748151 17142314

[29] S. T. Hess , T. P. Girirajan , M. D. Mason , Biophys. J. 2006, 91, 4258. 16980368 10.1529/biophysj.106.091116 PMC1635685

[30] R. Chen , X. Tang , Y. Zhao , Z. Shen , M. Zhang , Y. Shen , T. Li , C. H. Y. Chung , L. Zhang , J. Wang , B. Cui , P. Fei , Y. Guo , S. Du , S. Yao , Nat. Commun. 2023, 14, 1. 37202407 10.1038/s41467-023-38452-2 PMC10195829

[31] C. Arbore , L. Perego , M. Sergides , M. Capitanio , Biophys. Rev. 2019, 11, 765. 31612379 10.1007/s12551-019-00599-y PMC6815294

[32] I. A. Favre‐Bulle , E. K. Scott , Trends Cell Biol. 2022, 32, 932. 35672197 10.1016/j.tcb.2022.05.001 PMC9588623

[33] R. R. Brau , J. M. Ferrer , H. Lee , C. E. Castro , B. K. Tam , P. B. Tarsa , P. Matsudaira , M. C. Boyce , R. D. Kamm , M. J. Lang , J. Opt. A Pure Appl. Opt. 2007, 9, 8.

[34] B. E. Vos , T. M. Muenker , T. Betz , Curr. Opin. Cell Biol. 2024, 88, 102374. 38824902 10.1016/j.ceb.2024.102374

[35] W. Hardiman , M. Clark , C. Friel , A. Huett , F. Pérez‐Cota , K. Setchfield , A. J. Wright , M. Tassieri , Acta Biomater. 2023, 166, 317. 37137402 10.1016/j.actbio.2023.04.039

[36] H. Šípová , L. Shao , N. O. Länk , D. Andrén , M. Käll , ACS Photonics 2018, 5, 2168.

[37] L. Shao , Z.‐J. Yang , D. Andren , P. Johansson , M. Käll , ACS Nano 2015, 9, 12542. 26564095 10.1021/acsnano.5b06311

[38] M. Pelton , M. Liu , H. Y. Kim , G. Smith , P. Guyot‐Sionnest , N. F. Scherer , Opt. Lett. 2006, 31, 2075. 16770437 10.1364/ol.31.002075

[39] C. Selhuber‐Unkel , I. Zins , O. Schubert , C. Sönnichsen , L. B. Oddershede , Nano Lett. 2008, 8, 2998. 18720978 10.1021/nl802053h

[40] E. W. Ades , F. J. Candal , R. A. Swerlick , V. G. George , S. Summers , D. C. Bosse , T. J. Lawley , J. Invest. Dermatol. 1992, 99, 683. 1361507 10.1111/1523-1747.ep12613748

[41] K. Kamal , W. Du , I. Mills , B. E. Sumpio , J. Cell. Biochem. 1998, 71, 491. 9827695 10.1002/(sici)1097-4644(19981215)71:4<491::aid-jcb4>3.0.co;2-p

[42] R. A. Budworth , M. Anderson , R. H. Clothier , L. Leach , Toxicol. In Vitro 1999, 13, 789. 20654551 10.1016/s0887-2333(99)00052-1

[43] N. O. Länk , P. Johansson , M. Käll , ACS Photonics 2020, 7, 2405.

[44] D. Andrén , N. O. Länk , H. Šípová Jungová , S. Jones , P. Johansson , M. Käll , J. Phys. Chem. C 2019, 123, 16406.

[45] A. M. Smith , M. C. Mancini , S. Nie , Nat. Nanotechnol. 2009, 4, 710. 19898521 10.1038/nnano.2009.326 PMC2862008

[46] M. Dogterom , B. Yurke , Science 1997, 278, 856. 9346483 10.1126/science.278.5339.856

[47] F. Eghiaian , I. A. T. Schaap , Structural and Dynamic Characterization of Biochemical Processes by Atomic Force Microscopy, Humana Press, Totowa, NJ, USA, 2011, pp. 71–95. 10.1007/978-1-61779-261-8_6 21809201

[48] F. Hajizadeh , L. Shao , D. Andrén , P. Johansson , H. Rubinsztein‐Dunlop , M. Käll , Optica 2017, 4, 746.

[49] G. Balogh , I. Horváth , E. Nagy , Z. Hoyk , S. Benkõ , O. Bensaude , L. Vígh , The FEBS Journal 2005, 272, 6077. 16302971 10.1111/j.1742-4658.2005.04999.x

[50] E. Nagy , Z. Balogi , I. Gombos , M. Åkerfelt , A. Björkbom , G. Balogh , Z. Török , A. Maslyanko , A. Fiszer‐Kierzkowska , K. Lisowska , P. J. Slotte , L. Sistonen , I. Horváth , L. Vígh , Proc. Natl. Acad. Sci. USA 2007, 104, 7945. 17470815 10.1073/pnas.0702557104 PMC1876552

[51] G. Balogh , G. Maulucci , I. Gombos , I. Horváth , Z. Török , M. Péter , E. Fodor , T. Páli , S. Benkő , T. Parasassi , M. D. Spirito , J. L. Harwood , L. Vígh , PLoS ONE 2011, 6, e21182. 21698159 10.1371/journal.pone.0021182 PMC3116874

[52] C. Lissandrello , F. Inci , M. Francom , M. R. Paul , U. Demirci , K. L. Ekinci , Appl. Phys. Lett. 2014, 105, 11. 10.1063/1.4895132 PMC4187256 25316924

[53] J. Salvador , M. L. Iruela‐Arispe , Front. Cell Dev. Biol. 2022, 10, 10.1038/nrm.2016.153. PMC9247619 35784481

[54] R. Ungricht , U. Kutay , Nat. Rev. Mol. Cell Biol. 2017, 18, 229. 28120913 10.1038/nrm.2016.153

[55] D. A. Starr , H. N. Fridolfsson , Annu. Rev. Cell Dev. Biol. 2010, 26, 421. 20507227 10.1146/annurev-cellbio-100109-104037 PMC4053175

[56] L. Blanchoin , R. Boujemaa‐Paterski , C. Sykes , J. Plastino , Physiol. Rev. 2014, 94, 235. 24382887 10.1152/physrev.00018.2013

[57] J. M. Hausdorff , C.‐K. Peng , Phys. Rev. E 1996, 54, 2154. 10.1103/physreve.54.2154 9965304

[58] L. S. Tsimring , Rep. Prog. Phys. 2014, 77, 026601. 24444693 10.1088/0034-4885/77/2/026601 PMC4033672

[59] D. D. Nolte , Rep. Prog. Phys. 2024, 87, 036601. 10.1088/1361-6633/ad2229 38433567

[60] W. Feneberg , M. Westphal , E. Sackmann , Eur. Biophys. J. 2001, 30, 284. 11548131 10.1007/s002490100135

[61] B. Fabry , G. N. Maksym , J. P. Butler , M. Glogauer , D. Navajas , N. A. Taback , E. J. Millet , J. J. Fredberg , Phys. Rev. E 2003, 68, 041914. 10.1103/PhysRevE.68.041914 14682980

[62] J. Alcaraz , L. Buscemi , M. Grabulosa , X. Trepat , B. Fabry , R. Farré , D. Navajas , Biophys. J. 2003, 84, 2071. 12609908 10.1016/S0006-3495(03)75014-0 PMC1302775

[63] J. Nixon‐Abell , C. J. Obara , A. V. Weigel , D. Li , W. R. Legant , C. S. Xu , H. A. Pasolli , K. Harvey , H. F. Hess , E. Betzig , C. Blackstone , J. Lippincott‐Schwartz , Science 2016, 354, 6311. 10.1126/science.aaf3928 PMC6528812 27789813

[64] D. Mizuno , C. Tardin , C. F. Schmidt , F. C. MacKintosh , Science 2007, 315, 370. 17234946 10.1126/science.1134404

[65] L. Li , S. Chen , J. Zheng , B. D. Ratner , S. Jiang , J. Phys. Chem. B 2005, 109, 2934. 16851306 10.1021/jp0473321

[66] R. G. Willaert , P. V. Boer , A. Malovichko , M. Alioscha‐Perez , K. Radotić , D. Bartolić , A. Kalauzi , M. I. Villalba , D. Sanglard , G. Dietler , Sci. Adv. 2020, 6, eaba3139. 32637604 10.1126/sciadv.aba3139 PMC7314535

[67] D. Kweku , M. I. Villalba , R. G. Willaert , O. M. Yantorno , M. E. Vela , A. K. Panorska , S. Kasas , Front. Bioeng. Biotechnol. 2024, 12, 10.3389/fbioe.2024.1348106. PMC10955466 38515626

[68] Y. Peng , J. Zhang , W. Xue , W. Wu , Y. Wang , K. Mei , Y. Chen , D. Rao , T. Yan , J. Wang , Y. Cao , S. Wu , Q. Zhang , Nano Res. 2023, 16, 2672. 10.1007/s12274-022-4333-3 PMC9118818 35610981

[69] P. Karpinski , Adv. Opt. Mater. 2022, 10, 1.

