# Supplementary material for: Rotating Gold Nanomotors for High‐Resolution Mapping of Subcellular Nanomotions
Source: Small Methods. 2025 Oct 21;9(12):e01393. doi: 10.1002/smtd.202501393 (PMC12716197; doi:10.1002/smtd.202501393)
Supplement: Supplementary file 1 — Supporting Information [file SMTD-9-e01393-s003.pdf]

Supporting Information

Rotating Gold Nanomotors for High-Resolution Mapping of Subcellular Nanomotions

Emelie Tornéus,† Charlotte Hamngren Blomqvist,‡ Caroline Beck Adiels,‡ and Hana Šípová-Jungová*,†

†*Department of Physics, Chalmers University of Technology, 412 96 Göteborg, Sweden*

‡*Department of Physics, University of Gothenburg, 412 96 Göteborg, Sweden*

E-mail: hana.jungova@chalmers.se

Outline of the Supporting Information

- **S.1 Optical Setup**
- **S.2 Viability Assay**
- **S.3 Nanomotor Calibration**
  - S.3.1 Sensitivity Calculations
  - S.3.2 Asymmetry in Calibration Curves
  - S.3.3 Resolution of the Nanomotion Detection in Water and Cell Media
  - S.3.4 Nanomotion Detection Resolution
  - S.3.5 Short-Time Fourier Transform Analysis of Nanomotor Rotation
  - S.3.6 Measurements of Local Temperature
- **S.4 Cell Measurements**
  - S.4.1 Fixed HMEC-1 Cell
  - S.4.2 Nanomotion Speed in Cell 2 and Cell 3
  - S.4.3 Frequency Analysis of Nanomotions Using Power Spectral Density and Scaling Exponents
  - S.4.4 Cellular Nanomotion Analysis with Short-Time Fourier Transform
- **S.5 Supplementary Videos**
- **References**

S.1 Optical Setup

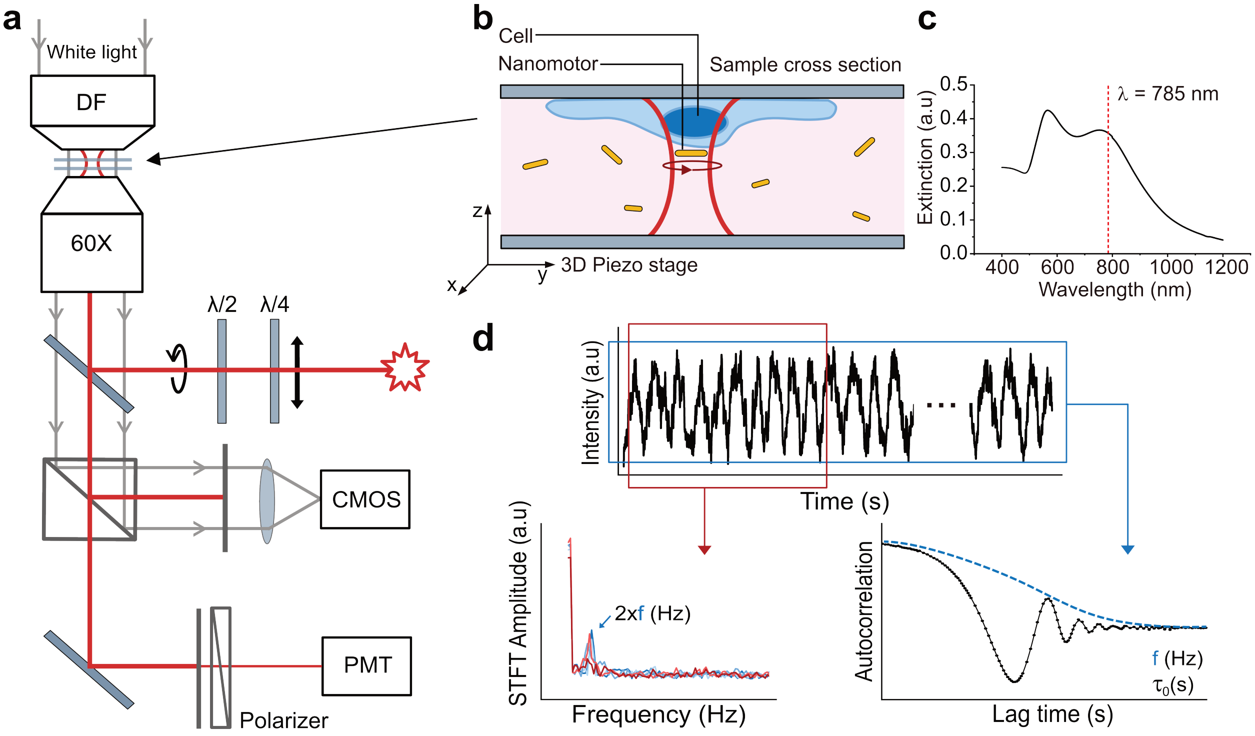

Figure S.1: (a) Schematic illustration of the optical setup: the inverted microscope enables trapping of a single gold nanorod against the upper cover glass of the sample chamber. Rotation of the nanorod is induced through spin angular momentum transfer, generated by a circularly polarized near-infrared laser beam ($\lambda = 785$ nm). The trapped particle is illuminated from above by a white light Dark-Field (DF) condenser. Laser light scattered from the particle passes through a dichroic beam-splitter and appropriate filters before being collected by a fiber-coupled photomultiplier tube (PMT), which is connected to a hardware correlator for particle movement analysis. The sample is mounted on a 3D piezo stage and visualized using an sCMOS camera. (b) Illustration of the sample cross-section, showing a cell adhered to the upper surface with a single nanomotor trapped over it and gold nanorods dispersed in the cell media. (c) Extinction spectra for nanorods with size $\sim 164 \times 98$ nm measeured with UV-Vis absorbtion spectrometer (Varian Cary). Red line indicates the wavelength of the trapping laser ($\lambda = 785$ nm). (d) Top: Intensity trace of backscattered photons from the nanomotor over time. Bottom left: Short Time Fourier Transform (STFT) showing a peak at twice the rotation frequency, $2f$. Bottom right: Autocorrelation function (ACF) of the recorded signal, yielding the rotation frequency and decay time τ_0.

S.2 Viability Assay

Nanorods were initially stabilized with CTAB after synthesis. Since CTAB-coated rods are known to be cytotoxic, we verified that nanorods functionalized with AT-EG_4-OH are biocompatible are biocompatible and do not affect cellular viability, even at concentrations 100× higher than those used in trapping experiments and during overnight exposure.

The nanorods (size $\sim 164 \times 98$ nm) were functionalized with AT-EG_4-OH following the established protocol. HMEC-1 cells were seeded at $\sim 50\%$ confluency in four 35 mm glass-bottom Petri dishes. The culture medium was supplemented with functionalized nanorods at a 1:100 dilution of the nanorod stock solution, corresponding to a concentration approximately 100× higher than that used in trapping experiments. Cells were then incubated overnight at 37 °C.

Viability was evaluated using the Live/Dead Viability/Cytotoxicity Kit for mammalian cells (L3224; Thermo Fisher Scientific) together with Hoechst nucleic acid stain (Thermo Fisher Scientific). The staining solution consisted of phosphate-buffered saline containing calcium and magnesium, PBS (+/+) (GE Healthcare, Sweden; containing Ca^{2+} and Mg^{2+}), supplemented with calcein-AM (1:1000 v/v), ethidium homodimer-1 (1:1000 v/v), and Hoechst (1:2000 v/v).

Prior to staining, the cells were rinsed with 2 mL PBS (+/+), followed by incubation with the staining solution for 15 min at 37 °C in the dark. Imaging was performed using a Leica fluorescence microscope (DMI6000B, Leica Microsystems, Wetzlar, Germany) with a 20× objective, allowing discimination of live (green), dead (red), and total (blue) cells. Assays were performed in PBS (+/+) rather than culture medium, as serum proteins in the media interfere with amine-reactive dyes.

For quantitative analysis, the images were processed using ImageJ (NIH, Bethesda, MD, USA). After applying channel-specific thresholds, the numbers of live (green, calcein-AM positive), dead (red, ethidium homodimer-1 positive), and total (blue, Hoechst positive) cells were determined. Cell viability was calculated as the percentage of live cells relative

to the total cell count using the formula:

$$Viability(\%) = \frac{all\ cells - dead\ cells}{all\ cells} \times 100$$

where *all cells* is the number of Hoechst-stained nuclei and *dead cells* is the ethidium homodimer-1 positive cells. Based on this analysis, the viability of untreated control samples was 95.4±1.90%, while samples incubated with functionalized nanorods showed a comparable viability of 94.1 ± 2.50%. The experiment was repeated once ($n = 2$).

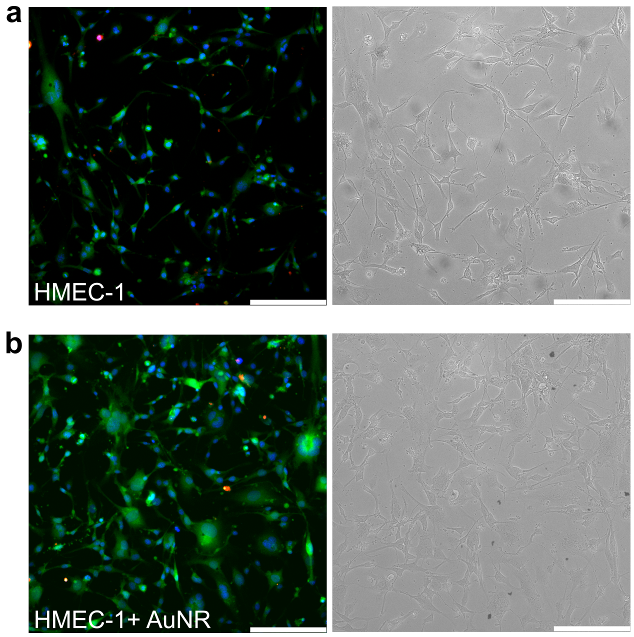

Figure S.2: Cell viability assay of the HMEC-1 cell line without (a) and with (b) the addition of gold nanorods functionalized with AT-EG_4-OH. Green fluorescence corresponds to live cells stained with calcein-AM, red fluorescence marks compromised or dead cells stained with ethidium homodimer-1 (EthD-1), and blue fluorescence highlights all nuclei stained with Hoechst. Phase-contrast images of the corresponding fields are shown on the right for structural context. Scale bar: 166μm.

S.3 Nanomotor Calibration

Calibration of the rotational frequency to axial displacement was performed by systematically changing the trapping height within the trapping beam using a piezo-controlled

stage (step size 100 nm). At each position, the rotational frequency was measured extracted from the back-scattered signal by autocorrelation function (ACF). Four measurements were performed at each position and the average value was used to construct the calibration curve. The resulting frequency–height relationship was fitted with a polynomial, providing calibration curves for direct conversion of frequency fluctuations into axial displacements. In Figure S.3, we plot the rotation frequency as a function of Δz, representing the change in axial position relative to the initial stage position z_0 (i.e., $\Delta z = z - z_0$). We note that z_0 is arbitrary and differs for each nanorod sample.

We report absolute rotational frequencies rather than normalizing to a maximum, because not every nanorod was calibrated across the full axial range and the true maximum frequency cannot be defined reliably. Instead, frequency changes were converted directly into axial displacements using the experimentally determined frequency–height relationship. This approach provides a straightforward mapping between frequency and position without requiring assumptions about the exact location of the laser focus.

Figure S.3(b) shows calibration curves obtained for several nanorods measured on different samples at the same laser power of 5 mW. When the curves are aligned to a common reference point corresponding to the same rotational frequency, they overlap closely, demonstrating that the calibration is consistent across measurements and independent of the individual nanoparticle or sample.

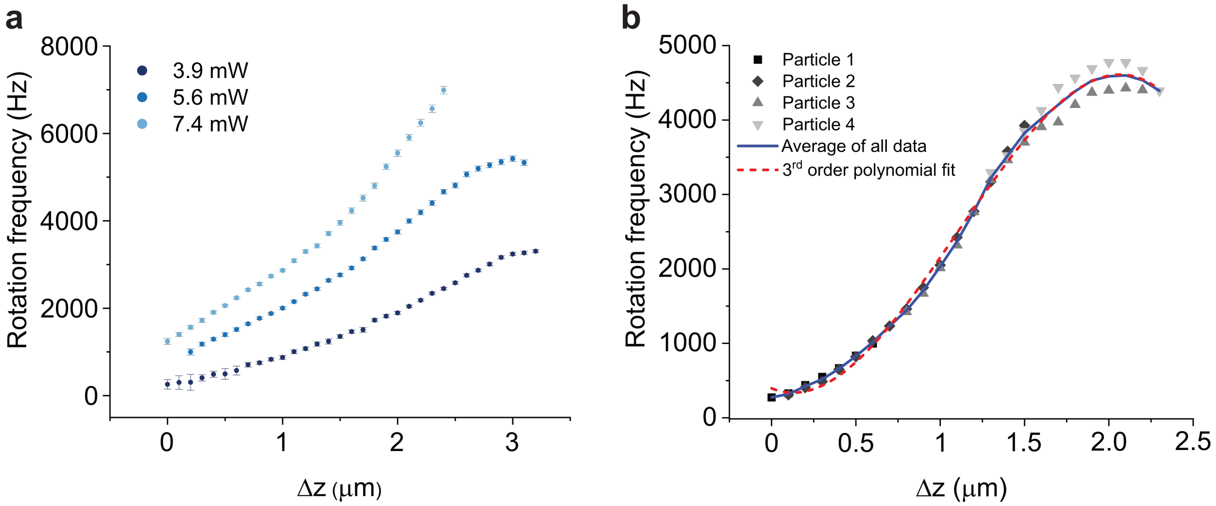

Figure S.3: (a) Rotation frequency as a function of axial position for nanorods of average size ∼164 × 98 nm, measured in water at three different laser powers: 3.9 mW, 5.6 mW, and 7.4 mW (b) Calibration curve constructed from four independent datasets obtained with different nanorods under identical conditions (cell medium, ∼5 mW, 100 nm axial steps). The averaged data were fitted with a 3rd-order polynomial, achieving an R^2 value of 0.99683. Absolute rotation frequencies are shown, demonstrating the reproducibility of the frequency–height relationship across nanorods. This calibration curve was used to convert rotation frequency into height for Cell 3 in Figure 3(b) of the main text. Data analysis was performed using ACF

S.3.1 Sensitivity Calculations

The relationship between rotation frequency (y) and displacement in z (x) is modeled using an nth-degree polynomial, typically a second-degree polynomial: $y = ax^2 + bx + c$. The sensitivity of the method is extracted from the slope of the polynomial, which is obtained from the derivative of the function. For a second-degree polynomial, the derivative is given by $\frac{dy}{dx} = 2ax + b$, while for a third-degree polynomial, it is $\frac{dy}{dx} = 3ax^2 + 2bx$. In cell medium, we observe a lower sensitivity compared to calibration curves made in water. This reduction is due to the higher viscosity of the medium and the non-specific binding of biomolecules to the particle surface, which increases the effective particle size and slows down its rotation. The calibration curve shown in Figure S.3(b) is fitted with a third-degree polynomial. Sensitivity is calculated from the inverse of the average

slope, which is 1.71 Hz/nm, resulting in a sensitivity of 0.5848 nm/Hz. We also extracted the sensitivity from seven individual calibration curves taken during cell measurements. The sensitivities from these calibration curves ranged between 0.2792 nm/Hz and 1.0125 nm/Hz, with an average sensitivity of 0.5458 nm/Hz.

S.3.2 Asymmetry in Calibration Curves

Figure S.3 shows the rotation frequency of a trapped nanorod as a function of axial position, revealing an asymmetry around the frequency maximum. This behavior is expected from the three-dimensional structure of the optical trap. In a tightly focused beam, the nanorod experiences maximal torque across a finite axial range, which produces the broad plateau in rotation frequency before the eventual decline on both sides. The asymmetry itself originates primarily from the axial force balance in optical tweezers. On one side of the focus, the scattering force reinforces the gradient force, whereas on the other side it counteracts it, resulting in an uneven force profile [1]. This, in turn, alters the distance between the surface and nanorod and the surface friction torque, causing a variation in rotation frequency above and below the beam focus [2]. This effect can be further exacerbated by spherical aberrations near the glass interface, which distort trap stiffness and symmetry [3]. In addition, high-NA focusing produces polarization-dependent focal fields that can bias the torque acting on anisotropic particles such as nanorods [4]. While these mechanisms provide a plausible explanation for the observed asymmetry, further experimental and theoretical studies are needed to fully elucidate their relative contributions. Moreover, the asymmetry could potentially be exploited to access two sensitivity regimes within the same trapping configuration.

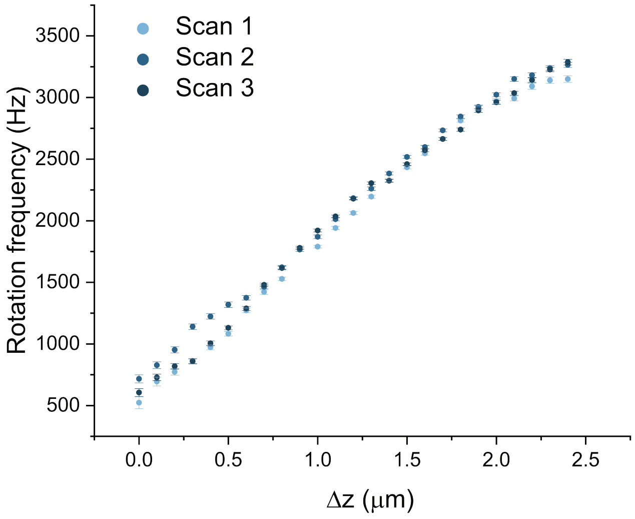

Figure S.4: The rotation of a single nanorod (approximately 164 × 98 nm in size) was measured along the optical axis using a 4 mW laser. Three separate scans were performed, each shown in different shades of blue, producing three calibration curves. The calibration curves show high sensitivity

S.3.3 Resolution of the nanomotion detection in water and cell media

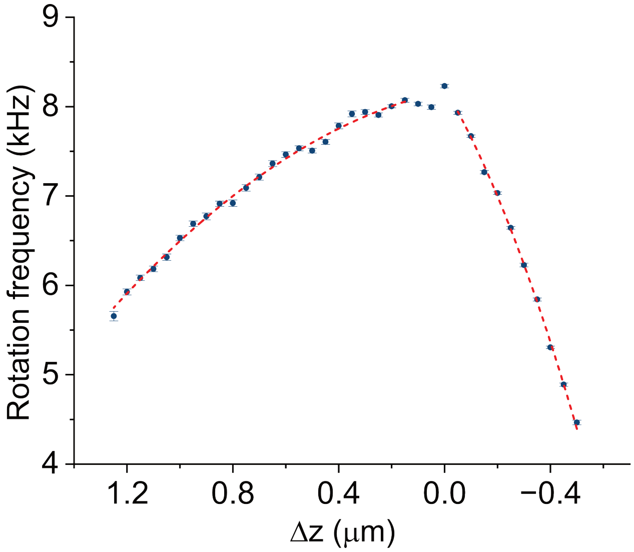

Figure S.5: Rotation frequency of an optically trapped nanorod (average size ∼135 × 70 nm) as a function of axial position of the nanopositioning stage. Two calibration branches are shown, corresponding to positions on either side of the maximum rotation frequency. The height is set to 0 at the point of maximum rotation frequency. Measurements were performed in water. Laser power: 7 mW.

S.3.4 Nanomotion Detection Resolution

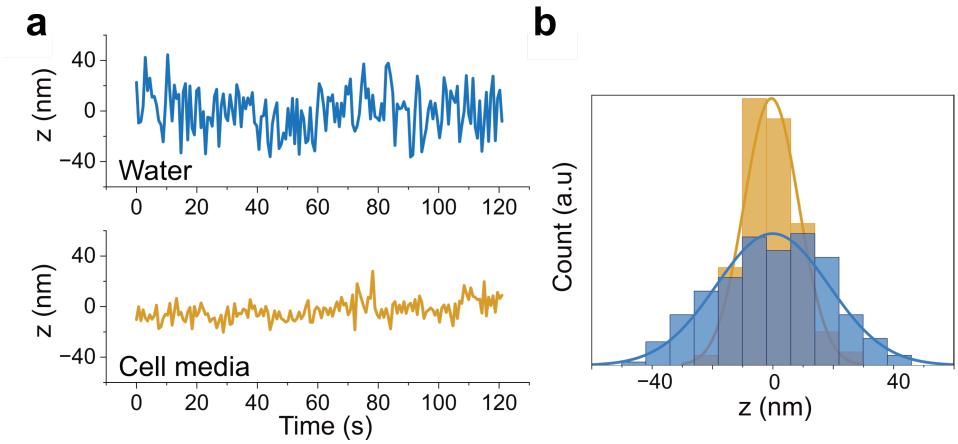

Figure S.6: (a) Height fluctuations of an optically trapped nanorod in water (blue) and cell media (yellow) over 120 seconds at a laser power of 5.5 mW. (b) Histogram of height positions for the nanomotors shown in (a). The standard deviation of the height fluctuations is approximately 20 nm in water and 10 nm in cell culture medium.

Comparison of Rotational and Intensity-Based Axial Tracking As intensity-based backscattering detection is a well-established method for tracking axial displacements in conventional optical tweezers, we compared it directly with our rotational-frequency approach. For the intensity readout, we used the total photon count acquired over a 0.5 s interval, while the rotation frequency was extracted from the same data using ACF analysis. For very small nanomotions, both methods yielded comparable resolution, with standard deviations of 18 nm (rotation-based) and 22 nm (intensity-based) for the measured amplitudes (Figure 2d, bottom). These results demonstrate that intensity detection can, in principle, resolve nanometer-scale fluctuations under favorable conditions. This similarity is most apparent in the low-amplitude regime

Figure 2c shows calibration measurements acquired in water, with each z-position measured five times. The resulting calibration curves, obtained with the two methods, display a similar dynamic range. However, the intensity-based data exhibited larger scatter of individual points around the calibration curve. To quantify calibration accuracy, we compared the axial position of the measured points obtained from the calibration curve

(z_{cal}) with the true position set by the piezo stage (z_{stage}). The difference between the two values was defined as the axial residual error (Δz_{res}):

$$\Delta z_{res} = z_{cal} - z_{stage}$$

This residual error quantifies the deviation of the calibration-derived position from the actual stage position and was used to evaluate the fidelity of the calibration curve. As shown on Figure 2c (bottom), the residual error of the intensity-based method fluctuated more strongly across different z-positions compared to the rotation-based. This behavior likely arises from interference effects and stray-light background, which vary with axial position and distort the scattering signal. As a result, the variable residual errors directly reduce the accuracy of nanomotion amplitude determination, leading to systematic over- or underestimation of displacement amplitudes. By contrast, the rotational-frequency method produced a more stable and reproducible calibration curves, with residual errors remaining low and consistent across larger axial range. This robustness makes the rotational approach particularly well suited for detecting large-amplitude cellular nanomotions, where interference strongly degrades intensity-based readouts.

S.3.5 Short-Time Fourier Transform Analysis of Nanomotor rotation

To capture the rapid dynamics of the nanomotor's rotation and improve the temporal resolution of our measurements, we applied Short-Time Fourier Transform (STFT) analysis to the backscattered photon data. This approach allows us to track fast nanomotions and extract the rotation frequency with high precision. The backscattered photons are collected with PMT at 100 kHz resulting in 50 000 data points for one 0.5-second measurement cycle. The data show periodic oscillation that correspond to the alignment of the long axis of the nanomotor with linear polarizer, i.e. there is scattering maxima every half-turn of the nanorod. By applying Short-Time Fourier Transfer (STFT) to this data

trace, we can increase the temporal resolution of nanomotion readout. In STFT the signal is divided into shorter segments on which the Fourier transform is computed. It is crucial that at least one full rotation of the nanomotor occurs within each time window (preferably more) to produce a distinct peak in the Fourier domain.

In Figure S.7 (a), we present the STFT amplitude for three measurements from a calibration curve taken at different positions along the z-axis. Time window was set to 2.56 ms, allowing for 2 - 20 full revolutions of the nanomotor during each window. In the STFT spectrogram, we observed a peak at twice the nanomotor's rotation frequency (Figure S.7 (a)). Figure S.7 (b) shows the frequency position of this peak over time, calculated as the location of the local maxima. By dividing this frequency by two, we obtain *f(t)* traces similar to the ones obtained with the ACF, but this time with time resolution on ∼2 ms. In Figure S.7(d), we compare the rotation frequencies along the optical axis extracted using STFT (averaged over 0.5 s) with those obtained from ACF analysis. Both techniques show a high level of agreement and can be used interchangeably, and our use of both methods is motivated by practicality: ACF is directly provided by the detector software for long recordings, whereas STFT is faster and more robust for short data segments where high temporal resolution is required.

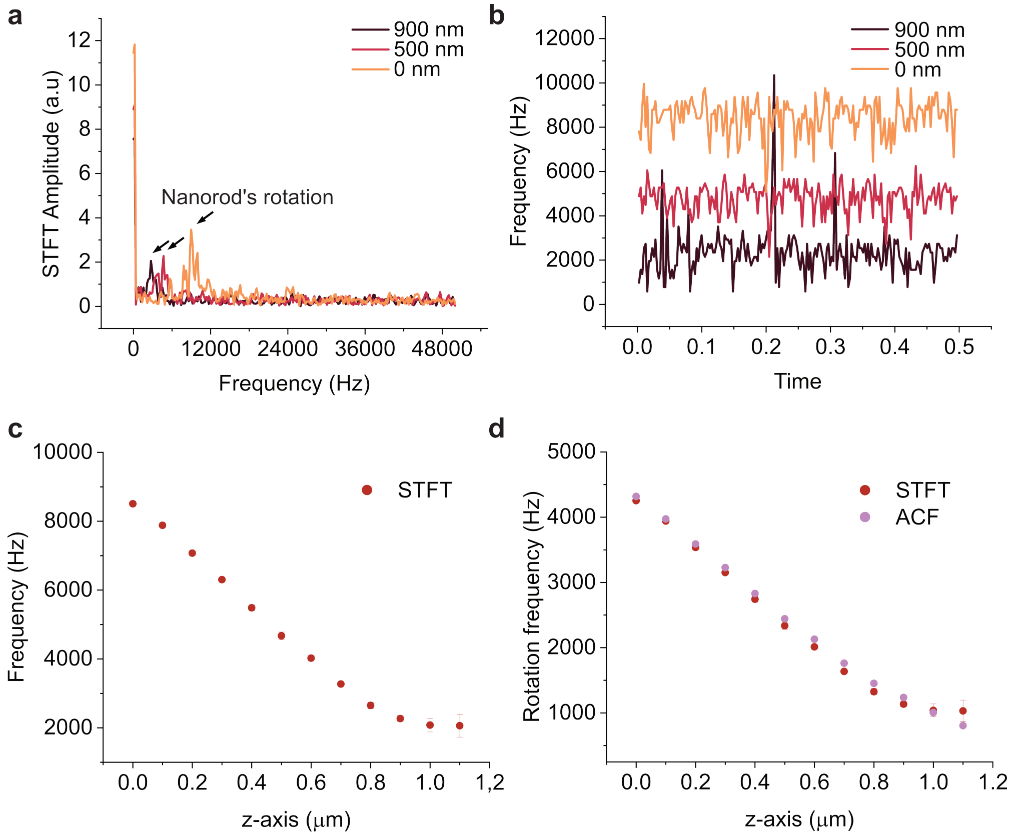

Figure S.7: (a) The STFT amplitude for one time window taken from three different position from a calibration curve; 0 nm, 500 nm and 900 nm. The backscattered photons from the rotating nanomotor results in a peak in the Fourier domain. (b) The frequency trace generated from the trapped nanomotor for one measurement cycle, for the three different positions. (c) The calibration curve from the STFT analysis, the frequency for each position is calculated from the mean frequency of one cycle and then the average from the mean values was extracted. (d) The STFT calibration curve divided by two to get the rotation frequency, compared with calibration curve generated from the ACF analysis.

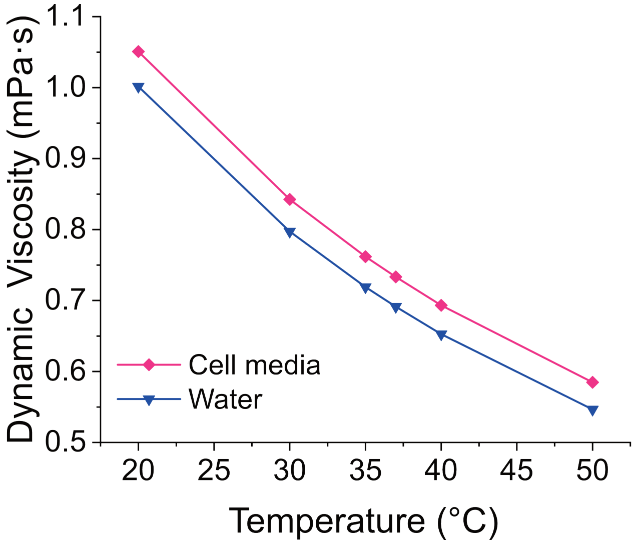

Figure S.8: The dynamic viscosity (mPa·s) for water (in blue) and HMEC-1 cell media (in magenta) is presented. The cell media were measured at the temperatures, 20, 30, 35, 37, 40 and 50 °C using the Lovis 2000 M microsviscometer from Anton Paar. The viscosity of water was obtained from measurement data provided on the Anton Paar website [5].

S.3.6 Measurements of Local Temperature

An additional consequence of variations in the beam profile is that the photothermal heating of the gold nanorod changes depending on its position along the optical axis. The autocorrelation function (ACF) decay time τ_0 is connected to the rotational friction coefficient γ_r and the effective rotational Brownian temperature T_r through $\tau_0 = \frac{\gamma_r}{4k_B T_r}$. The friction coefficient is given by $\pi\eta(T)gL^3$ where $\eta(T)$ represents the temperature-dependent dynamical viscosity of the surrounding water, g represents the geometrical shape factor dependent on the nanorod's eccentricity, and L is the length of the nanorod [6,7].

As a result, a trapped nanorod can be used as a local thermometer to measure the temperature in its immediate environment. In Figure S.9 we show the rotational Brownian temperature T_r as a function of nanorod location along the optical axis. The temperature increases almost linearly as the nanorod moves toward the laser focus and then levels off. This trend is observed consistently in both water and cell culture media.

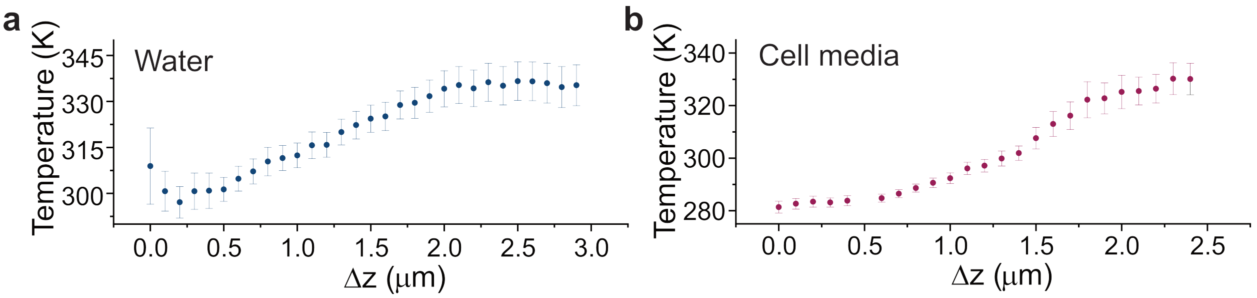

Figure S.9: (a) Local temperature of a trapped gold nanorod with an average size of approximately 168 × 98 nm. These measurements were conducted in milliQ-water using a laser power of 5.5 mW. (b) The same type of data as presented in (a), but with measurements performed in cell media, where the gold nanorod is functionalized with a monolayer of alkanethiols. Error bars represent the standard deviation of the fit for the temperature.

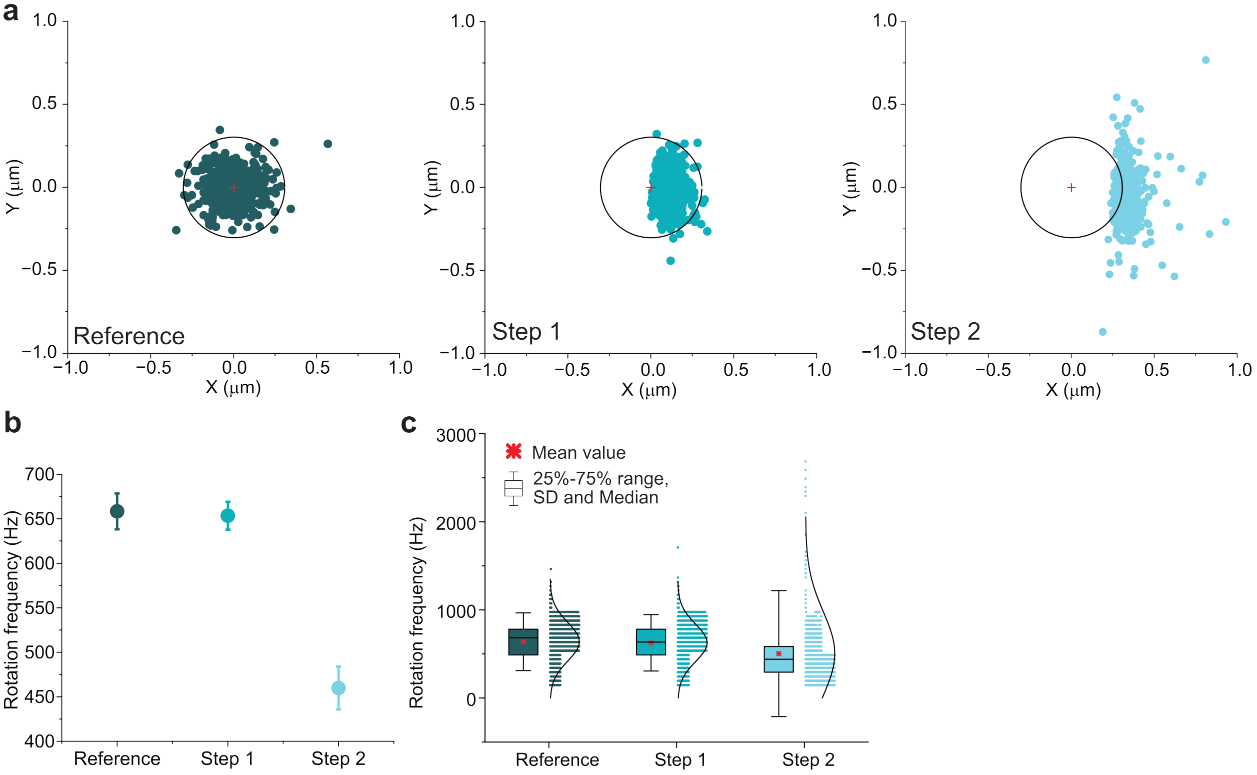

Figure S.10: (a) In-plane positions of the nanomotor observed using a CMOS camera (500 frames at 28 fps), with the beam center marked by a cross and a circle representing $3 \times \mathrm{SD}$ (0.3045 µm radius) of the in-plane positions of the reference. The particle is moved toward a 0.25 µm-high wall in consecutive steps. From left to right: (1) a reference measurement taken without the presence of the wall, (2) *Step 1*, where half of the laser beam is obscured by the wall, and (3) *Step 2*, where most of the beam is obscured. As the nanomotor approaches the wall, the optical trap becomes increasingly obscured, limiting the nanomotor's ability to access the full optical trap. (b) The nanomotor's rotation frequency at each step is shown, with error bars indicating the standard deviation from 30 measurements at each location. (c) The rotation frequency extracted using STFT. The distribution of rotation frequency is shown for the reference, Step 1, and Step 2. Box plots represent the inter-quartile range (25%-75%), with standard deviation (SD), median, and mean (red asterisk) highlighted for each data set. The nanomotors were functionalized with HS-$C_{11}EG_4OH$, and measured in 14.97 mM phosphate-buffered saline (PBS) using a 5 mW laser.

S.4 Cell Measurements

S.4.1 Fixed HMEC-1 Cell

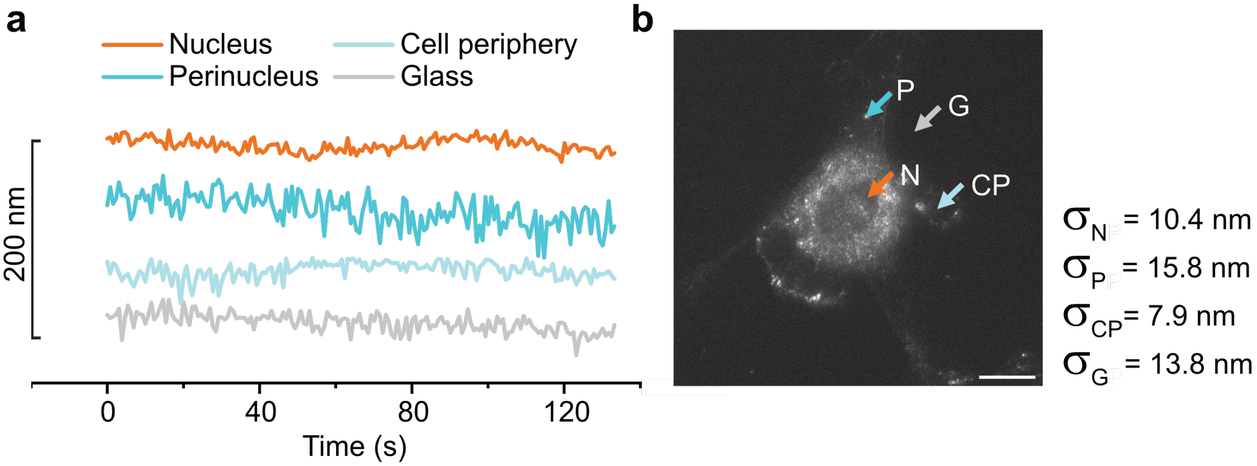

Figure S.11: (a) Nanomotion over time from three measurements taken over the nucleus (N), perinucleus (P), and cell periphery (CP) of a fixed HMEC-1 cell, along with one measurement over glass (G). All measurements were performed using a single nanomotor trapped with a laser power of 5.5 mW and analyzed using ACF. (b) Dark-field image of the fixed HMEC-1 cell with each measurement location indicated by arrows. The amplitude (σ) is shown for each measurement. Scale bar: 20 μm.

S.4.2 Nanomotion Speed in Cell 2 and Cell 3

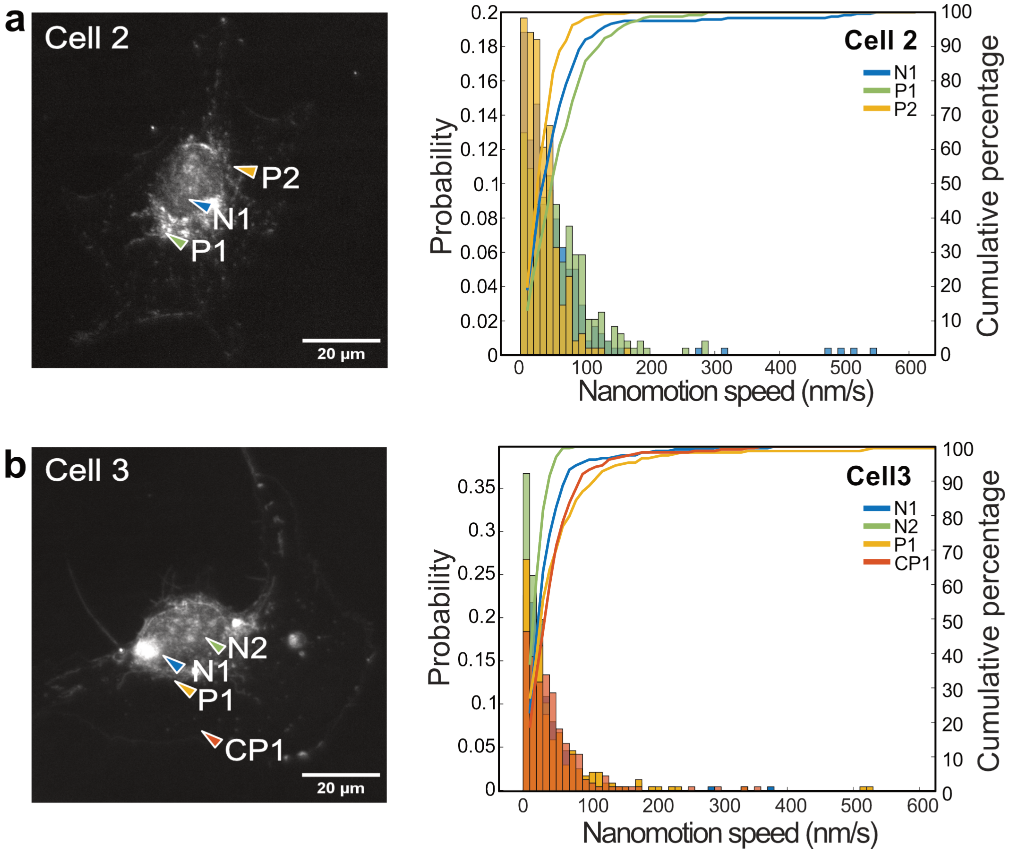

Figure S.12: Nanomotion speed for the Cells 2 and 3 cells that are analyzed in more detail in the main text and in Figure 5. Left: DFM images showing locations of the measurement probability distribution of nanomotion speed (nm/s) for the measurements made over Cell 2 (a) and Cell 3 (b) presented The histograms show change in height (nm) between each step z(n+1)-z(n) with a time period of 0.67 s. For Cell 3, a calibration curve was not made using the same nanorod that was used for the measurements, hence an average calibration curve (see Figure S.3(b)) was used to convert the rotation frequency into height.

S.4.3 Frequency Analysis of Nanomotions Using Power Spectral Density and Scaling Exponents

We analyze the z(t) trace obtained using the ACF function by calculating its Power Spectral Density (PSD) in the frequency range between 0.005 Hz and 0.7 Hz. The PSD is fitted with a scaling pattern described by C/f^{α}, where C adjusts the overall magnitude

of the PSD and α represents the scaling factor that provides insight into the degree of time-correlation in the measured nanomotions. An $\alpha \approx 0$ corresponds to white noise, indicating uncorrelated random processes. An $\alpha \approx 1$ is associated with pink noise, reflecting processes with some memory or correlation, and $\alpha > 1$ suggests red or Brownian noise connected to strong correlations and long-term memory in the system [8]. We also identify the knee frequency, which marks a transition in the PSD trend. The knee frequency is found at the intersection of two C/f^{α} fits: one applied to the lower frequency region, resulting in slope α_1, and the other to the higher frequency region, resulting in slope α_2. In some measurements, the PSD does not exhibit a change in across the measured frequency range, leading to the absence of a knee frequency in those cases.

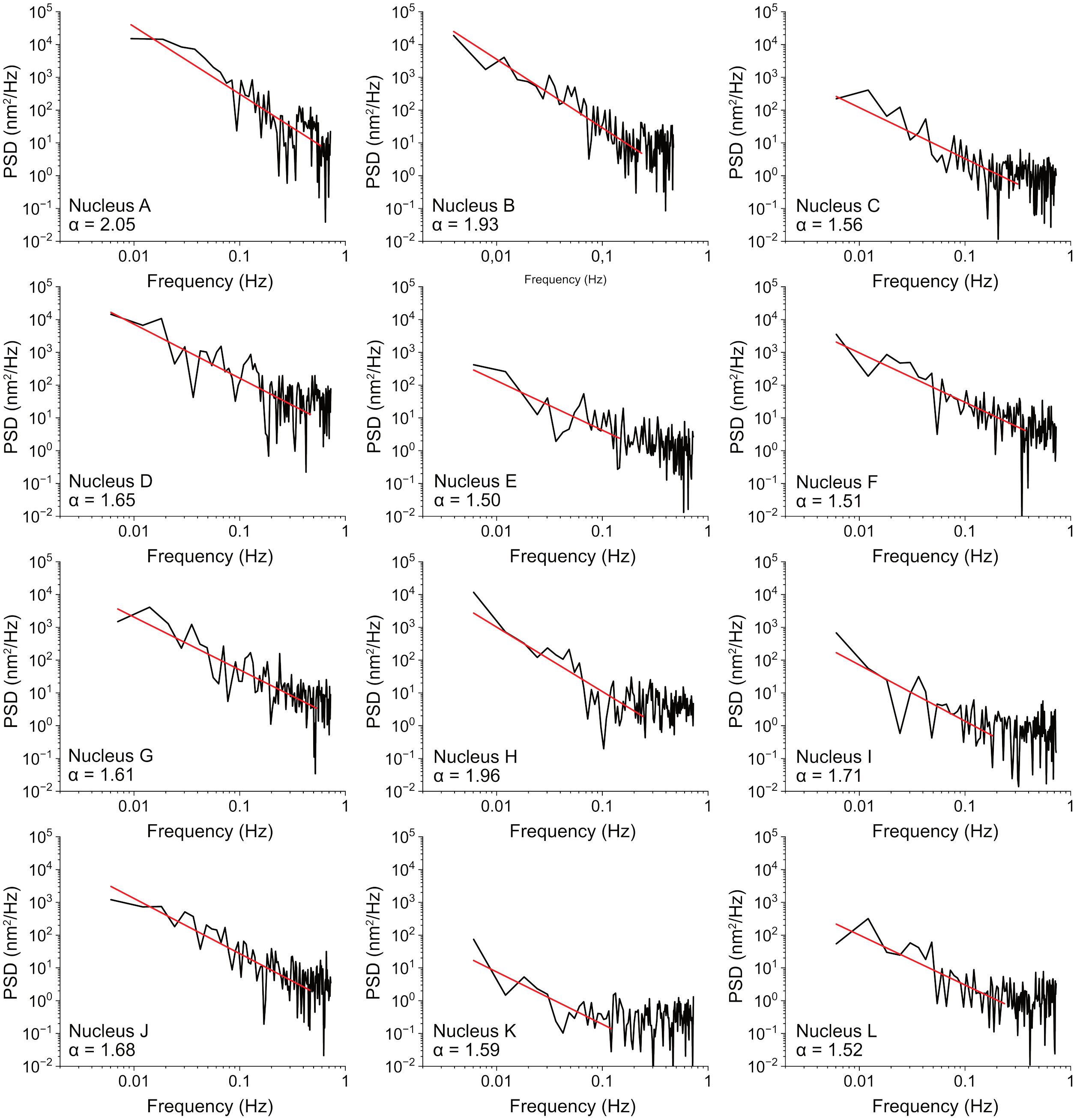

Figure S.13: Power Spectral Density (PSD) plots for all measurements taken at a cell nuclei. Measurement Nucleus B corresponds to the N1 measurement for Cell 2 in Figure 5 in the main text, while Nucleus H and Nucleus I correspond to the N1 and N2 measurements for Cell 3 in Figure 5. The $1/f^{\alpha}$ fit is shown in red along with the extracted α_1 for each data set.

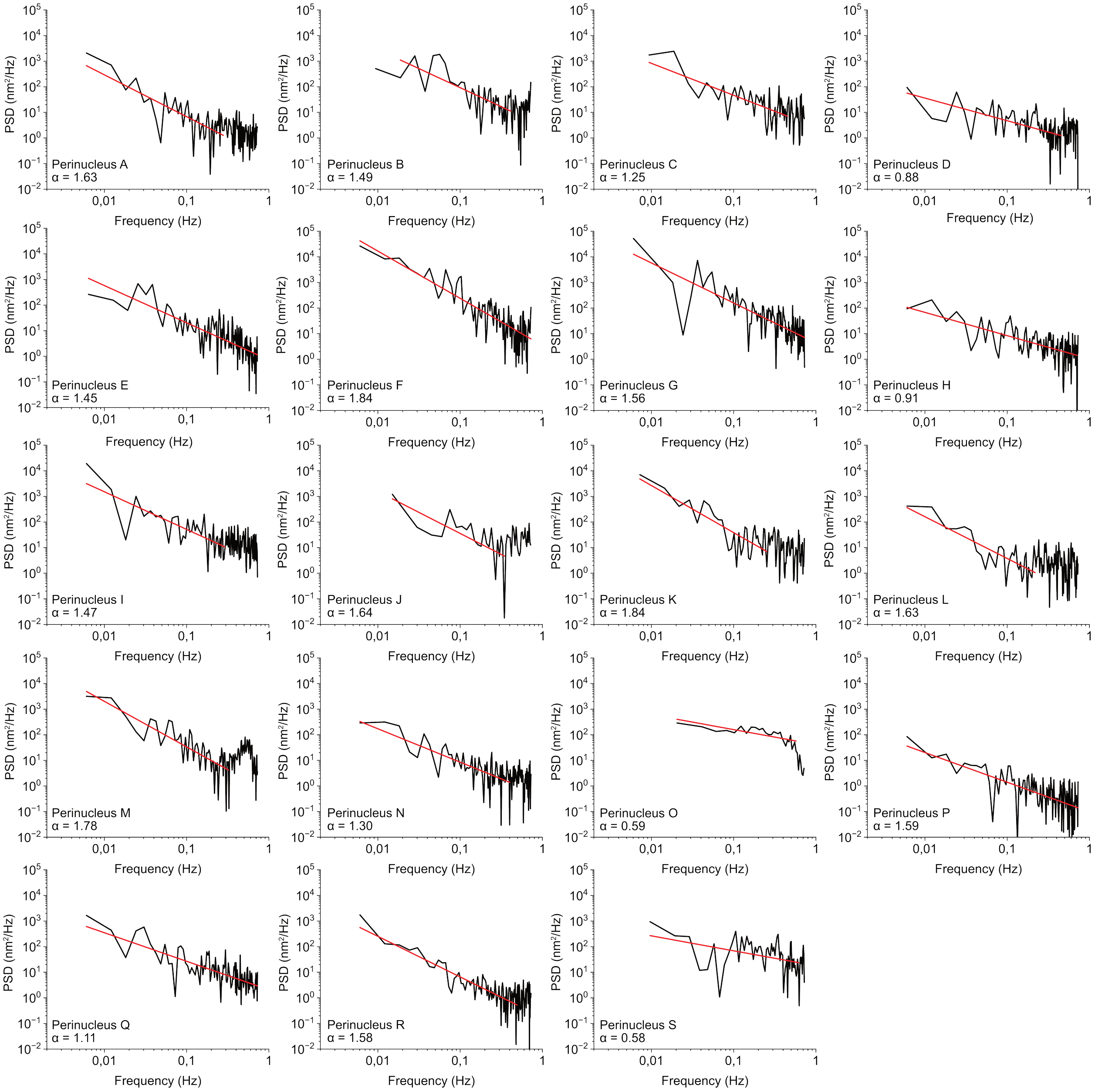

Figure S.14: Power Spectral Density (PSD) plots for all measurements taken at perinuclear regions. Measurement Perinucleus C and D are the P1 and P2 measurements for Cell 2 in Figure 5 in the main text, while Perinucleus M corresponds to the P1 measurement for Cell 3 in Figure 5. The $1/f^{\alpha}$ fit is shown in red, along with the extracted α_1 for each data set.

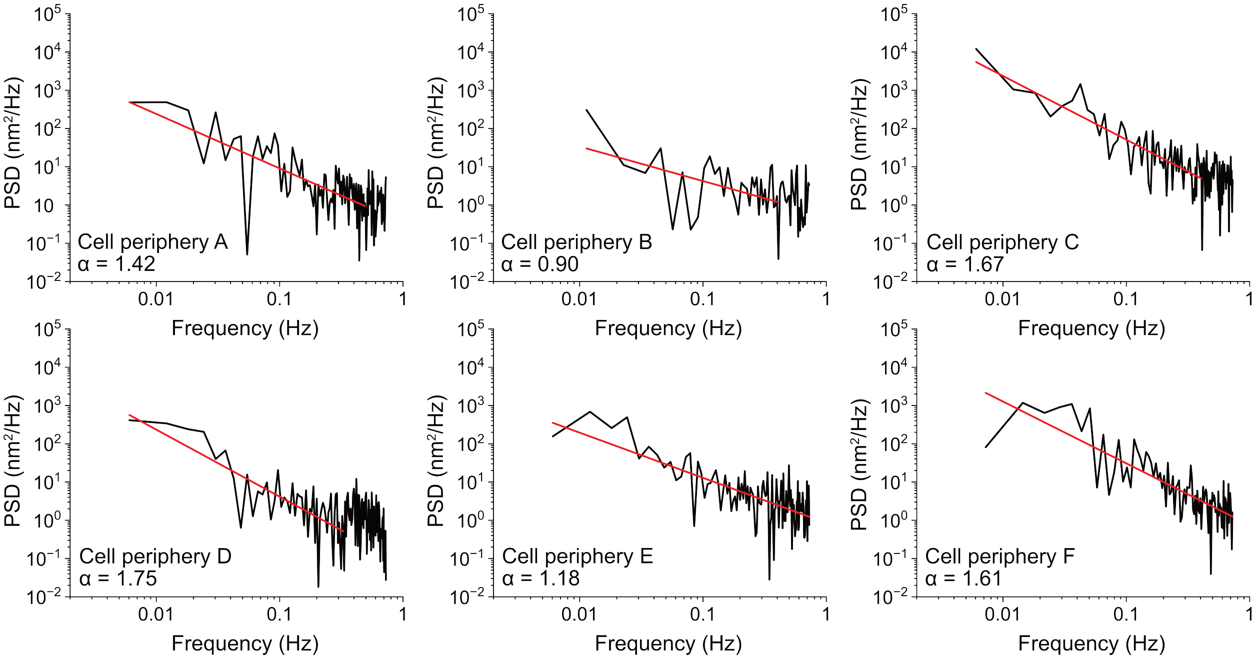

Figure S.15: Power Spectral Density (PSD) plots for each measurement made over the cell periphery. Here, measurement Cell periphery C is the CP1 measurement shown for Cell 3 in Figure 5 in the main text. The $1/f^{\alpha}$ relationship is shown in red together with the extracted α_1 for each data set.

S.4.4 Cellular Nanomotion Analysis with Short-Time Fourier Transform

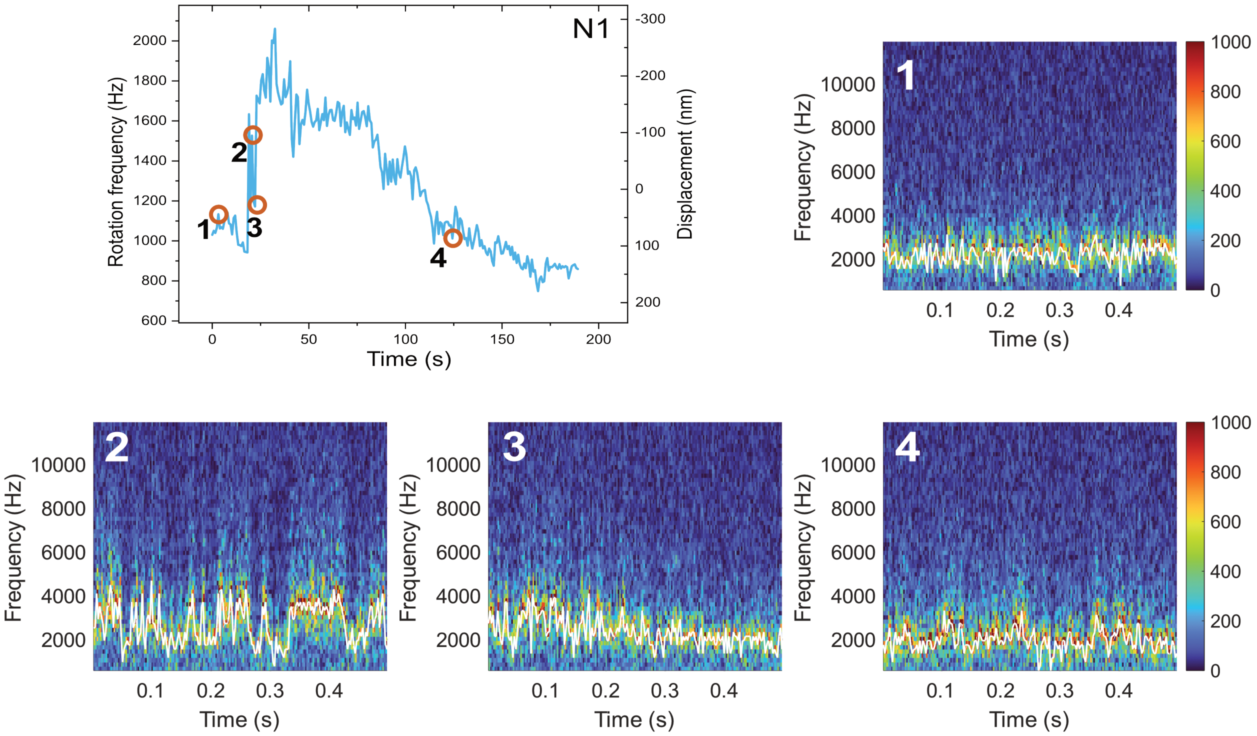

Figure S.16: Nanomotion recorded from the nuclear region of Cell 3, analyzed using the calibration curve shown in Figure S.3(b). STFT spectrograms derived from the backscattered intensity at selected time points 1,2,3 and 4, displaying a peak at 2f emphasized in white. The spectrogram illustrates the frequency fluctuations over time.

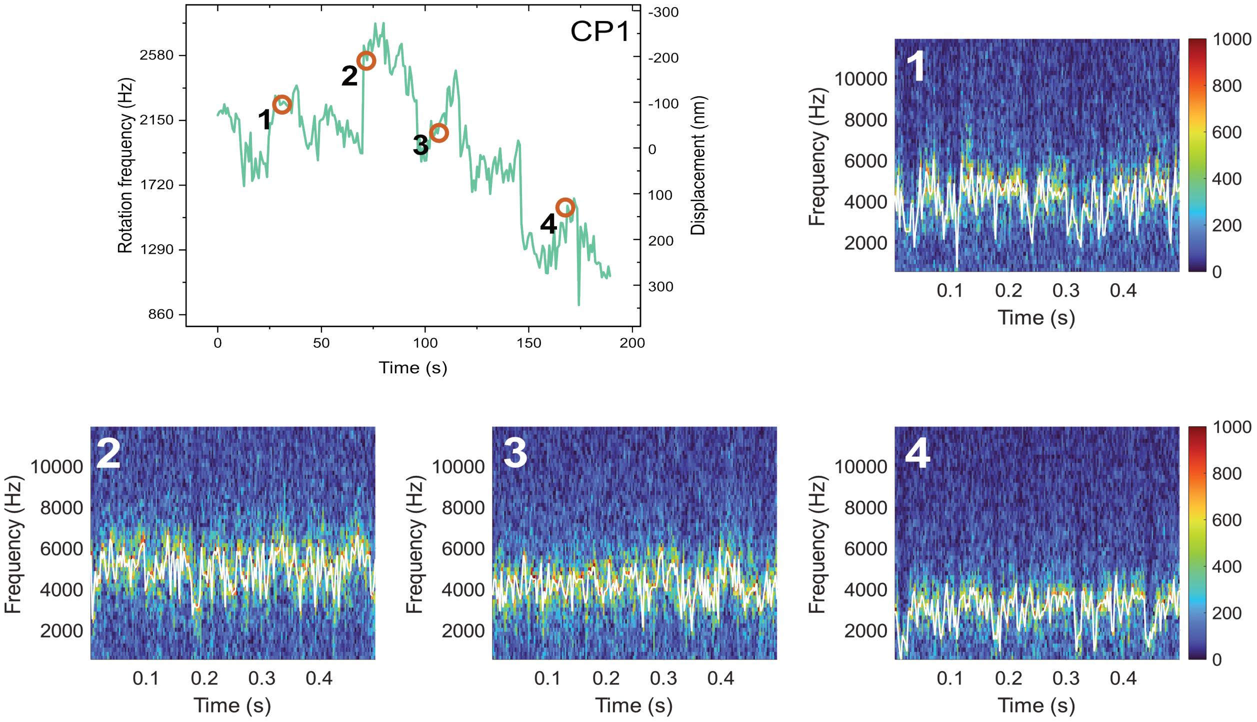

Figure S.17: Nanomotion recorded from the cell periphery region of Cell 3, analyzed using the calibration curve shown in Figure S.3(b). STFT spectrograms derived from the backscattered intensity at selected time points 1,2,3 and 4, displaying a peak at 2f emphasized in white. The spectrogram illustrates the rotation frequency fluctuations over time

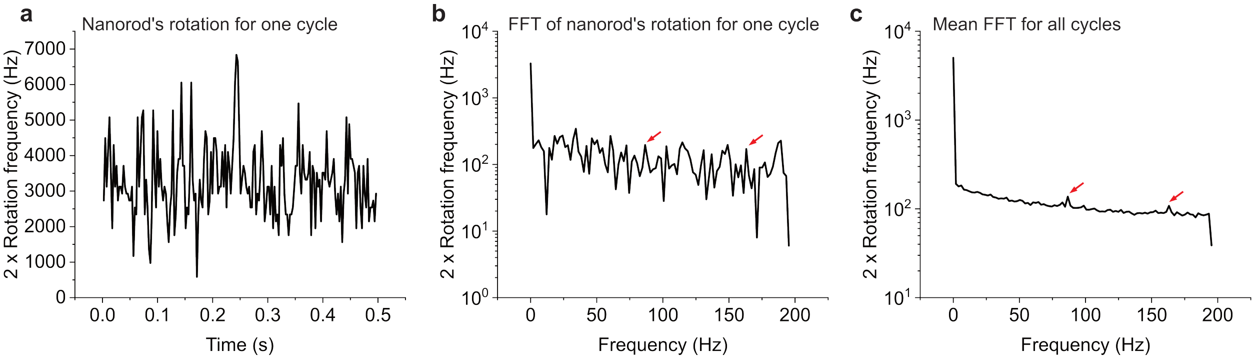

Figure S.18: (a) The frequency corresponding to the nanorod's rotation while trapped over glass, extracted from a STFT spectrogram, over one measurement cycle of 0.5 s. (b) The Fast Fourier transform of the frequency shown in (a). The red arrows indicate two distinct peaks resulting from environmental noise. (c) The mean FFT of all measurements cycles, with the red arrows indicating the peaks from environmental noise, here much more pronounced.

S.5 Supplementary Videos

The supplementary videos show the recorded motion of the trapped nanomotor during the measurements labeled N1, P1, and P2, as presented in Figure 3. Figure 3(c) displays the tracked in-plane position of the nanomotor over time, with color changes indicating temporal progression. Each video is named according to the corresponding measurement, FIG3_N1, FIG3_P1 and FIG3_P2. The recordings consist of 1000 frames captured at 5 frames per second.

References

[1] Hu, Y.; Nieminen, T. A.; Heckenberg, N. R.; Rubinsztein-Dunlop, H. Antireflection coating for improved optical trapping. Journal of Applied Physics 2008, 103, 9. https://doi.org/10.1063/1.2919574.

[2] Andrén, D.; Odebo Länk, N.; Šípová-Jungová, H.; Jones, S.; Johansson, P.; Käll, M. Surface interactions of gold nanoparticles optically trapped against an interface. The Journal of Physical Chemistry C 2019, 123 (26), 16406–16414.

https://doi.org/10.1021/acs.jpcc.9b05438.

[3] Rohrbach, A.; Stelzer, E. H. K. Trapping forces, force constants, and potential depths for dielectric spheres in the presence of spherical aberrations. Applied Optics 2002, 41, 13, 2494. https://doi.org/10.1364/AO.41.002494.

[4] Madadi, E.; Samadi, A.; Cheraghian, M.; Reihani, S. N. S. Polarization-induced stiffness asymmetry of optical tweezers. Optics Letters 2012, 37, 17, 3519. https://doi.org/10.1364/OL.37.003519.

[5] GmbH, A. P. wiki.anton-paar.com/be-en/water/. 2024.

[6] Šípová, H.; Shao, L.; Länk, N. O.; Andrén, D.; Käll, M. Photothermal DNA Release from Laser-Tweezed Individual Gold Nanomotors Driven by Photon Angular Momentum. ACS Photonics 2018, 5, 2168–2175.

[7] Hajizadeh, F.; Shao, L.; Andr´en, D.; Johansson, P.; Rubinsztein-Dunlop, H.; Käll, M. Brownian fluctuations of an optically rotated nanorod. Optica 2017, 4, 746–751.

[8] Hausdorff, J. M.; Peng, C.-K. Multiscaled randomness: A possible source of 1/f noise in biology. Physical Review E 1996, 54, 2154–2157.
